# Possible roles of phytochemicals with bioactive properties in the prevention of and recovery from COVID-19

**DOI:** 10.3389/fnut.2024.1408248

**Published:** 2024-07-10

**Authors:** Sachiko Koyama, Paule V. Joseph, Vonnie D. C. Shields, Thomas Heinbockel, Poonam Adhikari, Rishemjit Kaur, Ritesh Kumar, Rafieh Alizadeh, Surabhi Bhutani, Orietta Calcinoni, Carla Mucignat-Caretta, Jingguo Chen, Keiland W. Cooper, Subha R. Das, Paloma Rohlfs Domínguez, Maria Dolors Guàrdia, Maria A. Klyuchnikova, Tatiana K. Laktionova, Eri Mori, Zeinab Namjoo, Ha Nguyen, Mehmet Hakan Özdener, Shima Parsa, Elif Özdener-Poyraz, Daniel Jan Strub, Farzad Taghizadeh-Hesary, Rumi Ueha, Vera V. Voznessenskaya

**Affiliations:** ^1^School of Medicine, Indiana University, Indianapolis, IN, United States; ^2^Section of Sensory Science and Metabolism and National Institute of Nursing Research, National Institute of Alcohol Abuse and Alcoholism, Bethesda, MD, United States; ^3^Department of Biological Sciences, Fisher College of Science and Mathematics, Towson University, Towson, MD, United States; ^4^Department of Anatomy, Howard University College of Medicine, Washington, DC, United States; ^5^Indian Institute of Technology Ropar, Rupnagar, India; ^6^CSIR-Central Scientific Instruments Organisation, Chandigarh, India; ^7^ENT and Head and Neck Research Center and Department, The Five Senses Health Institute, School of Medicine, Iran University of Medical Sciences, Tehran, Iran; ^8^School of Exercise and Nutritional Sciences, San Diego State University, San Diego, CA, United States; ^9^VMPCT Private Practice, Milan, Italy; ^10^Department of Molecular Medicine, University of Padova, Padova, Italy; ^11^Department of Otolaryngology-Head and Neck Surgery, The Second Affiliated Hospital of Xi'an Jiaotong University, Xi'an, China; ^12^Department of Neurobiology and Behavior, University of California, Irvine, Irvine, CA, United States; ^13^Department of Chemistry, The Center for Nucleic Acids Science & Technology, Carnegie Mellon University, Pittsburgh, PA, United States; ^14^Department of Evolutionary Psychology and Educational Psychology, Universidad del País Vasco-Euskal Herriko Unibertsitatea, Leioa, Spain; ^15^Food Research Institute (IRTA), Monells, Spain; ^16^Severtsov Institute of Ecology & Evolution, Russian Academy of Sciences, Moscow, Russia; ^17^Department of Otorhinolaryngology, The Jikei University School of Medicine, Tokyo, Japan; ^18^Department of Anatomical Sciences, School of Medicine, Ardabil University of Medical Sciences, Ardabil, Iran; ^19^Monell Chemical Senses Center, Philadelphia, PA, United States; ^20^Transplant Research Center, Shiraz University of Medical Sciences, Shiraz, Iran; ^21^School of Pharmacy & Health Sciences, Fairleigh Dickinson University, Florham Park, NJ, United States; ^22^Department of Chemical Biology and Bioimaging, Wrocław University of Science and Technology, Wrocław, Poland; ^23^Swallowing Center, The University of Tokyo Hospital, Tokyo, Japan

**Keywords:** COVID-19, phytochemicals, foods, beverages, consumption habits, prevention, recovery

## Abstract

**Introduction:**

There have been large geographical differences in the infection and death rates of COVID-19. Foods and beverages containing high amounts of phytochemicals with bioactive properties were suggested to prevent contracting and to facilitate recovery from COVID-19. The goal of our study was to determine the correlation of the type of foods/beverages people consumed and the risk reduction of contracting COVID-19 and the recovery from COVID-19.

**Methods:**

We developed an online survey that asked the participants whether they contracted COVID-19, their symptoms, time to recover, and their frequency of eating various types of foods/beverages. The survey was developed in 10 different languages.

**Results:**

The participants who did not contract COVID-19 consumed vegetables, herbs/spices, and fermented foods/beverages significantly more than the participants who contracted COVID-19. Among the six countries (India/Iran/Italy/Japan/Russia/Spain) with over 100 participants and high correspondence between the location of the participants and the language of the survey, in India and Japan the people who contracted COVID-19 showed significantly shorter recovery time, and greater daily intake of vegetables, herbs/spices, and fermented foods/beverages was associated with faster recovery.

**Conclusions:**

Our results suggest that phytochemical compounds included in the vegetables may have contributed in not only preventing contraction of COVID-19, but also accelerating their recovery.

## 1 Introduction

The outbreak of coronavirus disease 2019 (COVID-19) became a long pandemic that lasted over 3 years. Although WHO declared that COVID-19 public health emergency ended on May 5, 2023 (1), there are still new variants of the SARS-CoV-2 prevailing and, in January 2024, there were more than 30,000 new patients weekly hospitalized in the U.S. (2). As of May 26, 2024, the cumulative cases of patients who contracted the virus surpassed 775 million with over 7 million deaths worldwide (3). Several factors may influence the infection, the severity of symptoms, and the recovery process, for example, genetic factors, age, and pre-existing conditions (4–9). Moreover, there were geographical differences in the number of cases around the world. It may have been the result of government strategies, such as herd immunity, and/or genetic differences (10, 11), however there was also the possibility of differences in the food consumed by different countries and the effects of phytochemical compounds included in them contributing to prevention of virus infection and/or improving the recovery process (12).

Indeed, multiple studies showed the effects of phytochemicals in plant-based diets and beverages on COVID-19 [e.g., tea (13, 14), plant-based diet (15, 16), the effects of eating carnation and citrus fruits (17)]. *In silico* and review studies have suggested that there are phytochemicals which may bind to the proteins of SARS-CoV-2 responsible for COVID-19 (18, 19). Such binding may contribute to suppress the infection if the phytochemicals bind to the spike protein of the virus (12, 20). Binding of the phytochemicals to the enzymes involved in the replication of the virus (12) may suppress the replication of the virus and may help recovery from COVID-19. Foods that contain large amounts of phytochemical compounds with bioactive properties may resemble the effects of drugs used for treatment of COVID-19 (21). These studies suggest that daily consumption of foods and beverages that contain these phytochemicals may prevent/suppress the contraction of COVID-19 or facilitate faster recovery from it (21–25). Some examples of these foods and beverages are green vegetables such as broccoli/cabbage, citrus fruits such as lemon and orange, red fruits such as strawberries and blueberries, and coffee and black tea.

In addition, some patients continue to have severe symptoms for long term after the acute phase of COVID-19 (post-acute sequelae of COVID-19 (PASC), or Long-COVID), and one of the reasons causing PASC is due to the remaining virus and particles of the virus in their tissues (26). Daily consumption of foods/beverages may help in their recovery by binding to the S-protein or the enzymes related to replication of these remaining virus or particles of the virus.

There are many phytochemicals with anti-inflammatory effects as well (19, 27). As inflammation is one of the key symptoms of COVID-19 and PASC, phytochemicals with anti-inflammatory effects may have beneficial influences on the recovery from COVID-19 and PASC (28, 29). Many of the phytochemicals included in cuisines possess anti-inflammatory and antioxidant effects [for example zingerone of ginger (30, 31) and curcumin of turmeric (30, 32)]. There are also phytochemicals that have anti-inflammatory effects as well as binding affinity to SARS-CoV-2 that causes COVID-19 [for example carvacrol in oregano and thyme (20, 33), cinnamaldehyde in cinnamon (33), geraniol in lemongrass, lavender, rose and others (33), curcumin in turmeric (33–35) and others (36)]. Depending on the traditional cuisines in each country, there may be differences in the type and amount of these phytochemicals consumed, and that may have contributed the differences in the infection, severity of the symptoms, and recovery from COVID-19. These phytochemicals are also included in beverages, such as teas from tea plants (14, 24) and herbal teas. A recent study on healthy identical twins has shown that those who were assigned to consume vegan diet had significantly improved cholesterol concentration, fasting insulin level, and body weight loss compared to those who were assigned to eat omnivorous food, indicating the effects of phytochemicals on several health conditions even among genetically identical twins (37). The health benefits of fermented plant-based foods have also been shown (38–40), which not only affect gut microbiota by the probiotic lactic acid bacteria in them, but also can improve the health status of the person directly by the phytochemicals with anti-inflammatory, antioxidant, and other bioactive properties included in them. Herbal medicines (41), supplements (42), and essential oils (43) may also contribute to prevent and improve the recovery from COVID-19 due to the phytochemicals with bioactive properties included in them.

The goal of the present study was to determine if there were associations between the self-reported consumption of phytochemicals and COVID-19 infection rates and recovery.

## 2 Methods

### 2.1 Participants

Data were collected from participants who consented to participate and were older than 18 years in age. Participation in this study was voluntary and was not remunerated, and informed consent was obtained from each participant at the beginning. The protocol was approved as an exempt study by Indiana University Human Research Protection Program (HRPP) in the U.S.A. (protocol #14915) as well as by the Office of Regulatory Research Compliance of Howard University (IRB-2022-0380), by the Jikei University School of Medicine Ethics Committee in Japan (#34–003), the Bioethics Committee at the A.N. Severtsov Institute of Ecology & Evolution of Russian Academy of Sciences (no.2022-63-NC), and by the Second Affiliated Hospital of Xi’an Jiaotong University, Medical Ethics Committee in China (#2022023). This study was conducted as an Internal Study of the Global Consortium for Chemosensory Research (GCCR),^1^ Project ID #NDS005, and invitation to participate in the study was also sent to participants of a previous study by GCCR, Project ID #GCCR001 (44). All methods were performed in accordance with the relevant guidelines and regulations in this study.

### 2.2 Survey development

We developed a global online survey in 10 different languages with questions related to consumption of different types and frequencies of the foods, beverages, herbal medicine, supplements, and essential oils people consumed or used topically or aromatically as home fragrance using a diffuser (see Supplementary Table S1 for questions in each language and Supplementary material for the English version of the survey). The participants had to access the survey via internet. We obtained information on demographic data, dietary habits, and on COVID-19 infection experiences. The link and the QR codes to the survey were distributed using social media such as Twitter, Facebook, etc., virtual communication platforms, flyers, and word of mouth. The questions focused on but were not limited to eating and drinking habits and frequency of intake (see Supplementary material for details). The questions on foods and beverages were multiple choice questions as well as free text questions so that participants could write the name of the foods and beverages that were not in the list of foods and beverage examples shown in the survey. We also asked whether a specific culture or a country influenced the answers to the questions on eating and drinking habits. Smoking habits and pre-existing conditions were also asked. Details on the survey development can be found in Supplementary material.

### 2.3 Processing and analyses of the results

Qualtrics software (Qualtrics, Provo, UT, United States)^2^ was used to obtain the raw data. All the data from each language version were then combined in one file to obtain the “Overall results” from all countries’ results (see the Results section). The time taken by participants to complete the survey was interpreted to represent their level of comprehension and accuracy in responding to questions. Therefore, data were excluded if the time to complete the survey was short. We arbitrarily selected 100s as the minimum time to finish the surveys. The participants who claimed to be younger than 18 years old were excluded. The participants who did not answer the question about whether they got COVID-19 were also excluded. Following this step, the distribution of selections of the frequency of eating and drinking (daily, weekly, monthly, never) and free text answers, depending on health condition (“Not tested,” “Tested positive,” and “Did not get COVID”), were analyzed.

Secondly, the data of each language version were analyzed separately. The answers concerning ethnicity and current location were analyzed to determine if each language version reflected any specific country and whether there was a specific country or culture influenced. Subsequently, the frequency (daily, weekly, monthly, never) of eating and drinking, and free text answers, depending on the health condition (“Not tested,” “Tested positive,” and “Did not get COVID”), were analyzed.

The datasets used and/or analyzed during the current study are available from the corresponding author on reasonable request.

### 2.4 Statistical analyses

We used Chi-square tests for comparisons of the food and beverage consumption, as well as the severity of symptoms. We used the Kruskal Wallis and Mann–Whitney U-tests to compare the days to recover from COVID-19. Systat 13.2 (Inpixon HQ, Palo Alto CA, United States) was used for the statistical analysis.

## 3 Results

### 3.1 Overall results (all countries together)

#### 3.1.1 Number of participants used for data analyses

The survey was conducted online. Overall, there were 1777 participants. About 10% of the participants (*n* = 187) completed the survey within 100 s and were excluded. Participants who were younger than 18 as well as the participants who did not answer the question on whether they contracted COVID-19 were also excluded. This made the amount of data from 1,583 participants eligible to be used for further analysis.

#### 3.1.2 Age and gender of the participants

The gender and age of the participants whose data were used in the analyses were male [average age 40.27 ± 14.60; *n* = 237 (26.87%)], female [average age 42.43 ± 14.07; *n* = 624 (70.75%)], non-binary/third gender [average age 37.50 ± 13.96; *n* = 4 (0.454%)], and prefer not to answer [average age 41.82 ± 12.70, *n* = 17 (1.51%)].

#### 3.1.3 COVID-19 status of the participants

The participants were asked about their COVID-19 status and to select from three categories, i.e., “Not tested but suspected,” “Tested positive,” and “Did not get COVID.” Of the 1,583 participants, 312 participants (19.7%) reported to not have been tested but suspected that they contracted COVID-19, 835 participants (52.7%) reported that they had tested positive for the virus, and 436 participants (27.5%) reported to have not contracted COVID-19 or contracted it unknowingly.

#### 3.1.4 Symptoms

**Figure 1:**
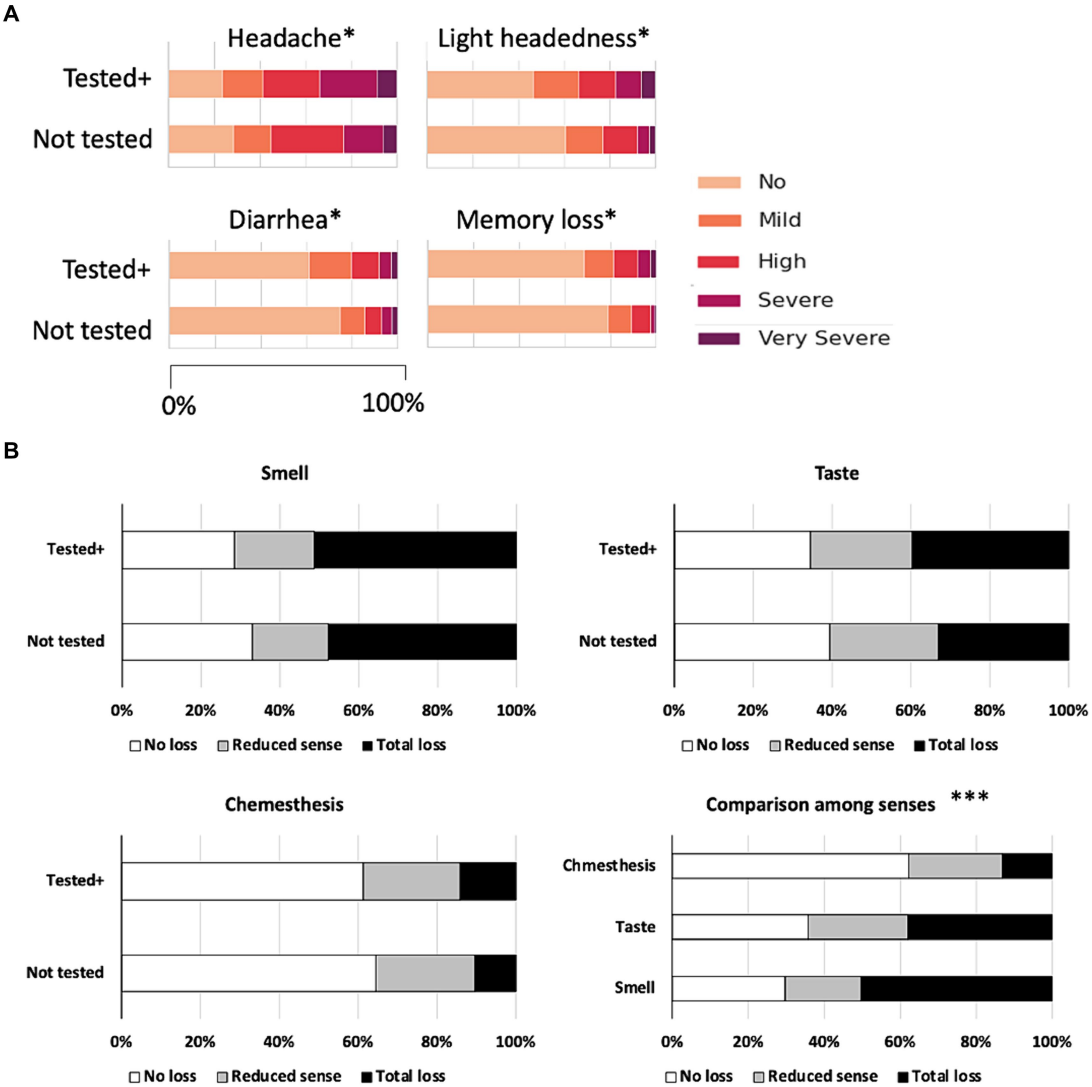
**(A)** Symptoms that showed significant differences between the “Not tested” and “Tested positive” groups. *Chi-square test, *p* < 0.05. **(B)** Self-reported senses of smell, taste, and chemesthesis by the “Not tested” and “Tested positive” groups and comparison of the occurrences of chemosensory dysfunction in the sense of smell, taste, and chemesthesis. ***Chi-square test, *p* < 0.001.

Overall, the participants of the “Tested positive” group reported more severe symptoms than the “Not tested” group especially in the symptoms of “Headache,” “Lightheadedness,” “Diarrhea,” and “Memory loss” (Figure 1A; Supplementary Table S2). There were no significant differences in other symptoms asked in the survey, such as fever, chills, body aches, and so on between the “Not tested” and “Tested positive” groups (Supplementary Figure S1; Supplementary Table S2).

Considering that the impairment of chemical senses is known to be one of the major symptoms of COVID-19, we compared the self-reported dysfunction in the chemical senses. Dysfunction in the senses of smell and taste were reported more frequently compared to chemesthesis, and there were no significant differences between the “Not tested” and “Tested positive” groups (Figure 1B). There were also no differences between the “Not tested” and “Tested positive” group in the dysfunction of chemesthesis (Figure 1B; Supplementary Table S2). Occurrences of chemosensory dysfunction of smell and taste (“Not tested” and “Tested positive” combined) were not largely different from each other, and dysfunction in chemesthesis was reported significantly less than the expected values. The differences in the occurrences of chemosensory dysfunction among the three types of senses overall were statistically significant (Figure 1). Thus, the sense of smell and taste are more vulnerable to COVID-19 compared to chemesthesis.

#### 3.1.5 Intake of foods and beverages

Based on the goal of this survey to determine whether there are differences in the consumption of each type of beverages and food materials, we focused on the differences between the “Tested positive” group and others, especially with “Did not get COVID” but also with “Not tested” group since the symptoms were lighter in this group compared to the “Tested positive” group.

##### 3.1.5.1 Beverages

**Figure 2:**
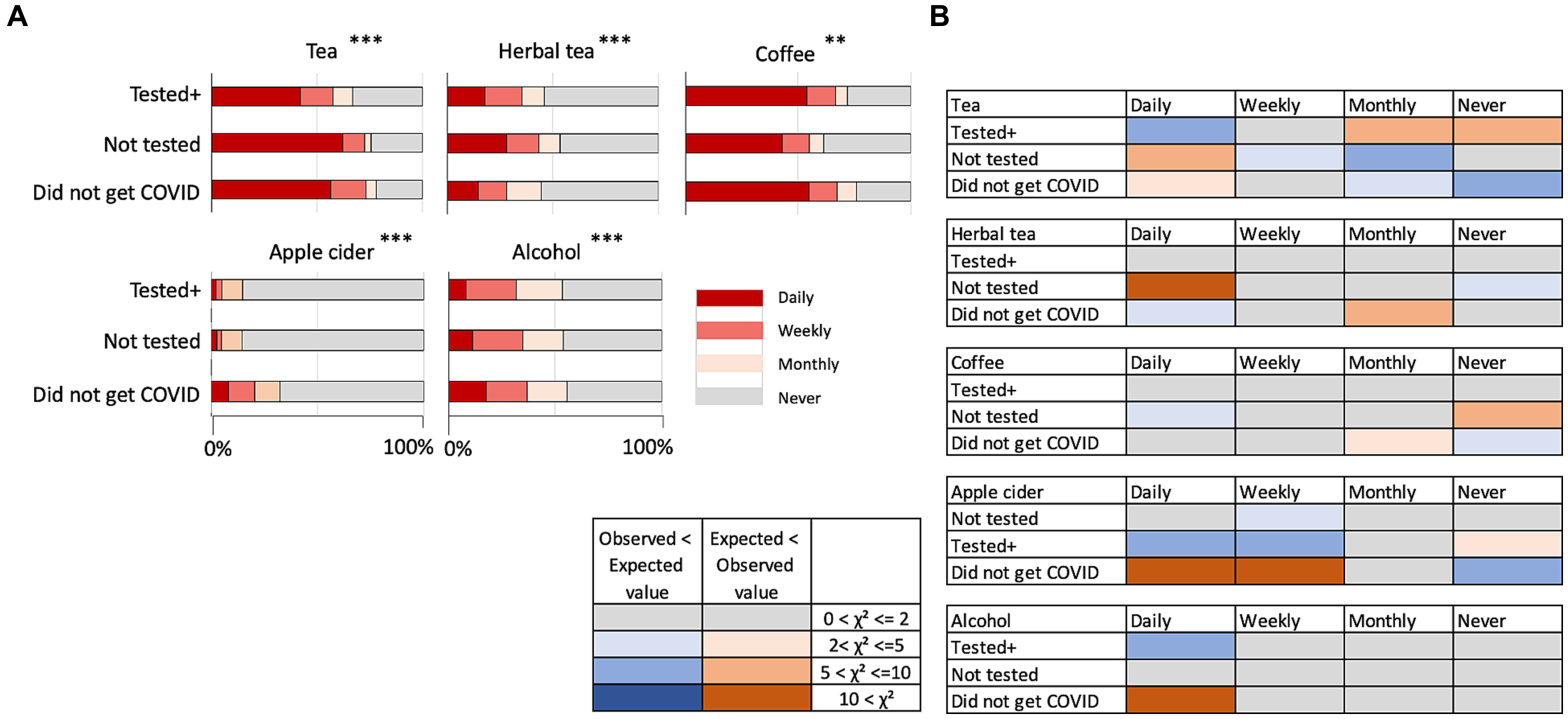
**(A)** Intake of tea, herbal tea, coffee, apple cider, alcohol, and hot chocolate. **p < 0.01, ***p < 0.001. **(B)** Discrepancy of observed values from expected values in the chi-square test results on beverages. The colors indicate that the observed values were higher than the expected values (smaller to larger discrepancy indicated by pink to red color) and the observed values lower than expected values (smaller to larger discrepancy indicated by light to dark blue). Gray color indicates there were no or negligible discrepancies.

Figure 2A shows the results on beverage consumption that showed significant differences among the three COVID status groups. Other than hot chocolate, we found significant differences among the three groups of participants (see Supplementary Table S3 as well). “Tea” and “coffee” were, in general, consumed daily more than other types of beverages.

Figure 2B shows whether the observed values were higher or lower than the expected values in each pair-wise comparison, contributing to the results of statistical significance in the Chi-square tests. “Tea” was significantly more daily consumed by participants who “Did not get COVID” and “Not tested.” Although herbal tea is often discussed with its beneficial effects on health, herbal tea was significantly less consumed by the “Did not get COVID” group. Apple cider was found to be significantly more consumed by “Did not get COVID” group participants, although the overall consumption was much less than other beverages. Coffee did not show specific tendencies among groups. Daily consumption of alcohol reported was higher in the “Did not get COVID” group. Hot chocolate was not consumed so frequently by any of the groups (data not shown; numbers and statistical results are shown in Supplementary Table S3).

##### 3.1.5.2 Foods

**Figure 3:**
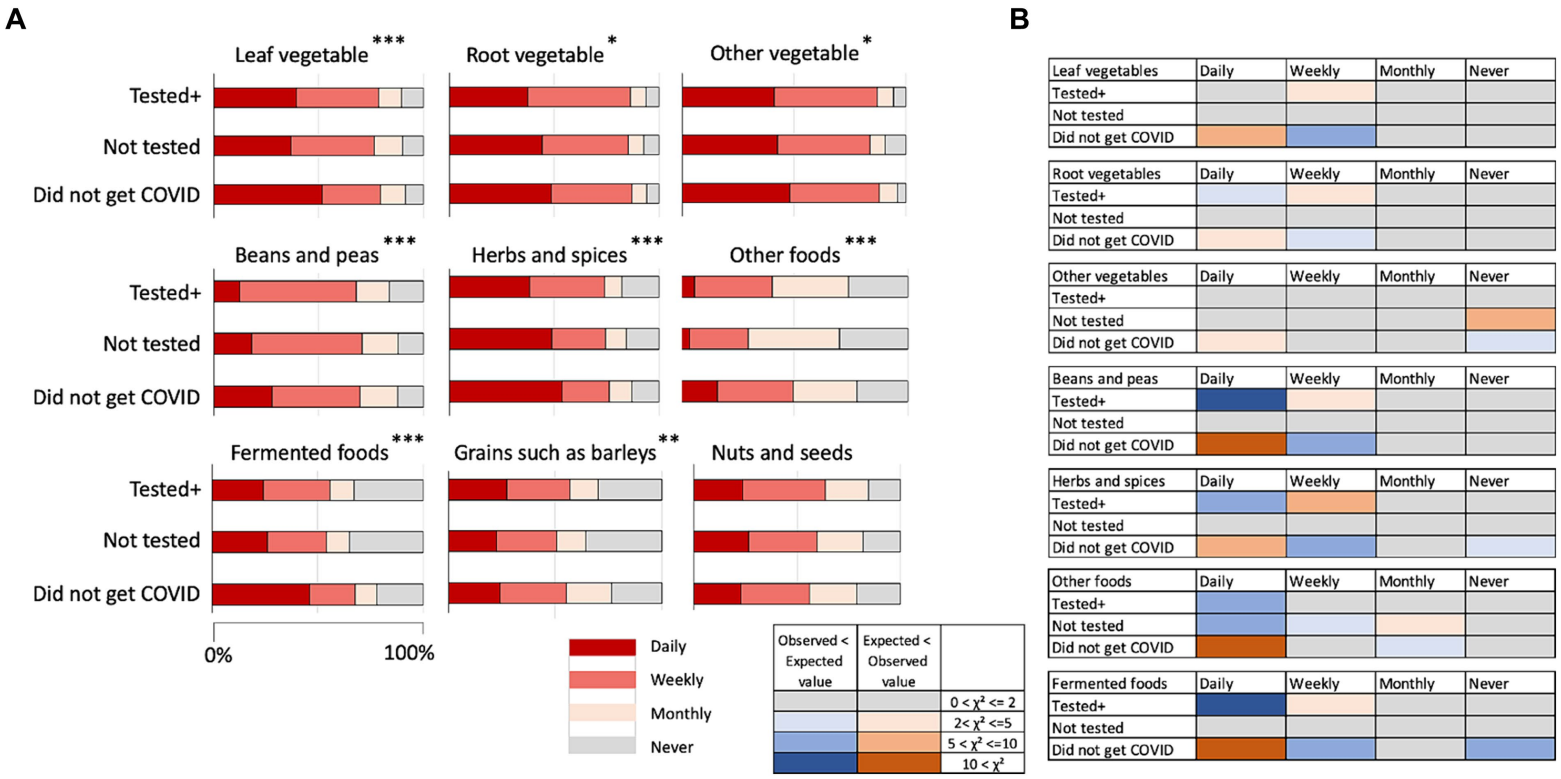
**(A)** Frequency of consuming various types of foods by the three groups. *p < 0.05, **p < 0.01, ***p < 0.001. **(B)** Discrepancy from expected values in the chi-square test results on foods. The colors indicate that the observed values were higher than the expected values (smaller to larger discrepancy indicated by pink to red color) and the observed values lower than the expected values (smaller to larger discrepancy indicated by light to dark blue). Gray color indicates there were no or negligible discrepancies.

There was an overall tendency that the “Did not get COVID” group daily consumed leaf, root, other vegetables, beans and peas, nuts and seeds, spices and herbs, other foods, such as mushrooms, and fermented food significantly more than the expected values compared to the other two groups (Figure 3A; Supplementary Table S3). Differences from the expected values showing that the “Did not get COVID” group daily consumed more compared to the other two groups were especially large in leaf vegetables, beans and peas, herbs and spices, other foods, and fermented foods (Figure 3B).

**Figure 4:**
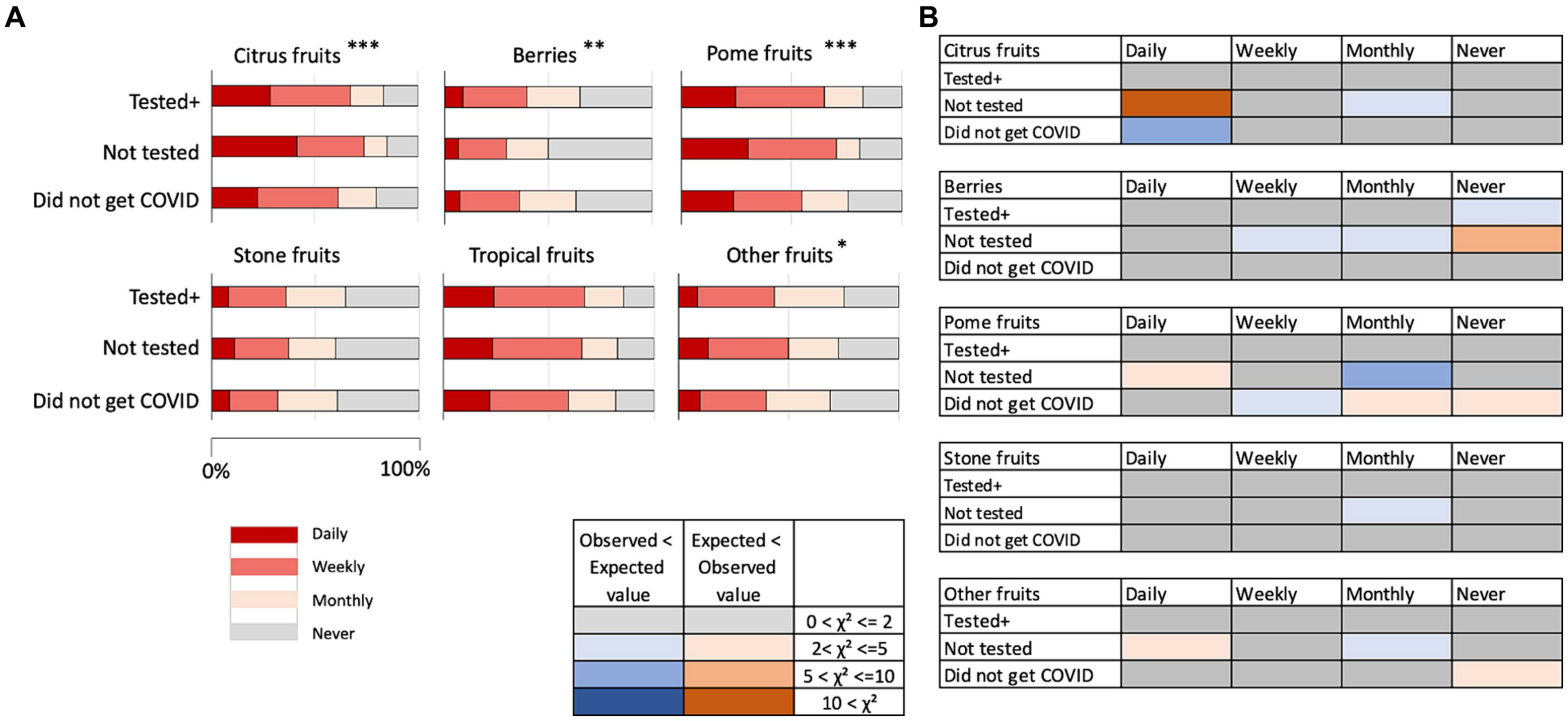
**(A)** Frequency of consuming fruits by the three groups. *p < 0.05, **p < 0.01, ***p < 0.001. **(B)** Discrepancy from expected values in the chi-square test results on fruits. The colors indicate that the observed values were higher than the expected values (smaller to larger discrepancy indicated by pink to red color) and the observed values were lower than the expected values (smaller to larger discrepancy indicated by light to dark blue). Gray color indicates there were no or negligible discrepancies.

In fruits, the consumption of citrus fruits, berries, pome fruits, stone fruits, and other fruits showed statistically significant differences in the frequency of eating among the health conditions of the participants (Figure 4A; Supplementary Table S3). Differences from the expected values, however, did not indicate that the “Did not get COVID” group daily ate fruits more than other groups (Figure 4B).

##### 3.1.5.3 Herbal medicines, supplements, and essential oils

There were significant differences in the way that herbal medicines and supplements were taken, and essential oils were used by the three groups (Supplementary Figure S2A; Supplementary Table S3). Comparison of the Chi-square scores revealed that the “Did not get COVID” group took herbal medicines and supplements or used essential oils significantly less than the expected values (Supplementary Figure S2B).

### 3.2 Comparison of countries

Next, we conducted analyses of the results for all the versions of languages separately. This was based on our interest in the cultural differences in the eating and drinking habits and their possible relationship with the infection rate in various countries, which became often discussed on social media at the beginning of the pandemic. The nationality of the participants of each language version of the survey did not necessarily mean that they were ethnically affiliated to a certain country (for example, Spanish is spoken in many countries). We determined the ethnicity of the participants in each language version of the survey using the answers to the questions on ethnicity and residing location. We found that, other than the English version, over 90% of the participants of each language version of the survey were ethnically affiliated to one country (Supplementary Table S4). Although Spanish is spoken in many countries, the participants in this survey were over 90% affiliated to Spain. The participants of the Persian version were also over 90% affiliated to Iran. This suggests that it is possible to expect that the results of each language version, other than the English version, may be able to represent geographical and cultural differences in the eating and drinking habits. The participants of the English version reported their ethnicity as Greek, Asian, Indian, Vietnamese, Israeli, Irish/German, Bosnian, and Montenegrin (Supplementary Table S4).

As the number of participants for some of the language versions of the survey was too few to analyze the data, we focused on the results of the language version that had more than 100 participants and excluding the English version because of the mixed ethnicity nature. Thus, the comparison of countries was conducted using the data of the Indian English (*n* = 122), Italian (*n* = 264), Japanese (*n* = 233), Persian (*n* = 245), Russian (*n* = 269), and Spanish (*n* = 252) versions of the survey to represent the six countries of India, Italy, Japan, Iran, Russia, and Spain.

#### 3.2.1 Beverages

**Figure 5:**
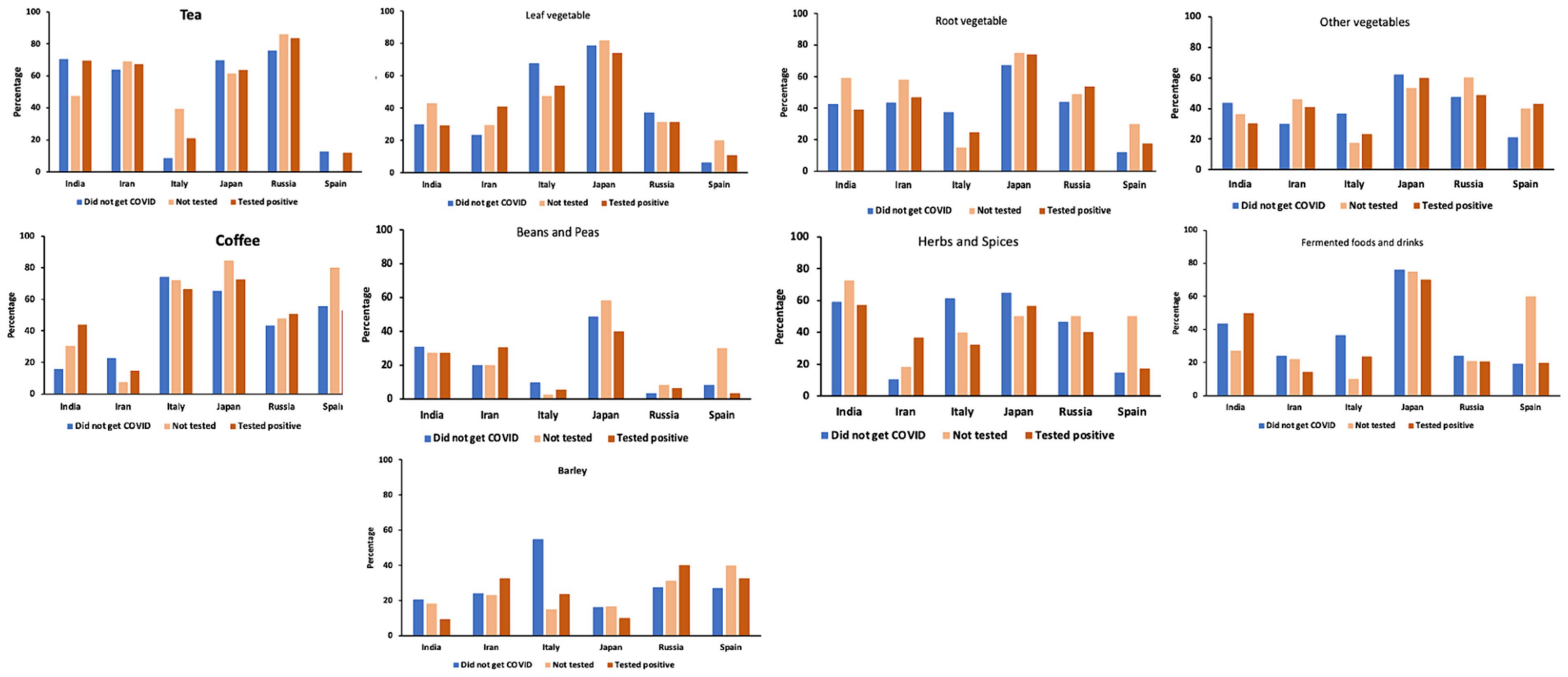
Percentage of participants in each group who have been drinking tea or coffee daily and the participants who were daily eating leaf vegetables, root vegetables, fermented foods, herbs and spices, beans and peas, and other foods such as mushrooms and olives.

We focused on the percentage of participants who reported that they have been drinking the type of beverage daily. We could see a large difference among the countries in the results. In Japan, the percentage of participants who reported to drink tea or coffee daily was similarly high (Figure 5). India, Russia, and Iran showed a high percentage of drinking tea daily but much less percentage of drinking coffee daily (Figure 5). Contrarily, in Italy and Spain, the percentage of drinking coffee daily was much higher than that of tea (Figure 5). Although the cultural differences were clear and suggested there might be differences in the tendencies of consumption if we take these differences into consideration, the percentages of daily consumption of tea and coffee among the health conditions did not show specific tendencies that suggest the benefits of tea or coffee to prevent contracting COVID-19.

**Figure 6:**
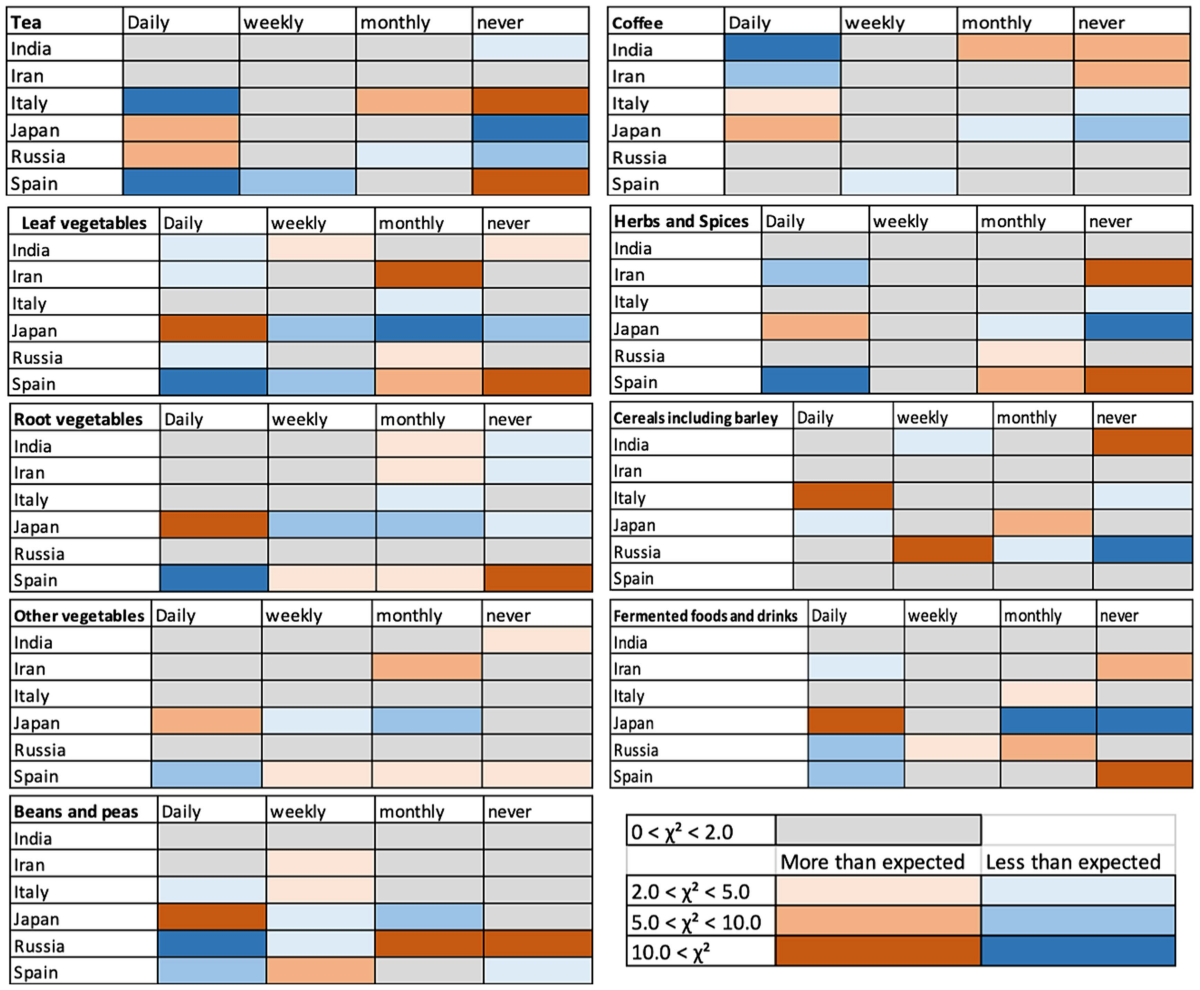
Discrepancy from expected values in the chi-square test results - comparison among the “Did not get COVID” group of each country. The colors indicate where the observed values were higher than the expected values (smaller to larger discrepancy indicated by pink to red color) and where the observed values were lower than the expected values (smaller to larger discrepancy indicated by light to dark blue). Gray color indicates there were no or negligible discrepancies.

Figure 6 shows the discrepancy between the expected values and observed values (Chi-square scores) of the “Did not get COVID” group of the six countries. Japan and Russia showed higher than expected values in the answers of “Daily” in tea, whereas Italy and Japan showed higher than expected values in the answers of “Daily” in coffee. The observed value of “Daily” in coffee by participants in Spain was not higher than the expected values but the observed value of “Daily” in tea was much lower than the expected value in Spain, suggesting that tea is not favored in Spain. These results also indicate the drinking habits of both tea and coffee in Japan, drinking coffee in Italy, and drinking tea in Russia.

#### 3.2.2 Foods

In the overall section, the results of leaf vegetables, root vegetables, fermented foods, beans and peas, cereals and grains including barley, and herbs and spices showed higher percentages of daily consumption by the “Did not get COVID” group. When the results were split among countries, we found large differences in the tendencies among them (Figure 5). Overall, Japan showed higher intake of all the categories of foods other than cereals and grains including barley, compared with other countries but there were no statistically significant differences among the health conditions other than the results on herbs and spices (Figure 5; Supplementary Figure S3 for the rest of the categories). The results by the “Did not get COVID” group of Italy showed clearly higher percentages of daily consumption in the seven categories of foods although the overall percentages were smaller than the results in Japan (Figure 5).

Figure 6 shows the discrepancy between the expected values and observed values in the answers by the “Did not get COVID” group of the six countries. The “Did not get COVID” group of Japan showed higher daily consumption of leafy vegetables, root vegetables, other vegetables, beans and peas, herbs and spices, and fermented foods, and Italy showed a higher consumption of cereals and grains including barley (Figure 6). The “Did not get COVID” group of Spain showed a lower daily consumption of leaf vegetables, root vegetables, other vegetables, beans and peas, herbs and spices, and fermented foods, showing a clear contrast to the results of the “Did not get COVID” group of Japan.

Such differences among countries also suggest the possibility that, in the results where there were no clear differences among health conditions in the overall analysis section above, the differences depending on the country might have compensated each other and produced that lack of tendencies. Indeed, there were large differences in the percentages of participants who ate the food types daily that did not show specific tendencies in the overall section depending on countries and health conditions (Supplementary Figure S3). Interestingly, the results of the survey in Japanese showed low percentages of daily consumption of all the types of fruits regardless of the health condition type, compared to other countries (Supplementary Figure S3). Considering the high percentage of daily consumption in all types of vegetables in the Japanese version, “daily consumption of vegetables and less frequent consumption of fruits” seemed to be the characteristics of food consumption in Japan. The results of the Italian version of the survey showed higher daily consumption of tropical fruits, barleys, other fruits, and other vegetables by the “Did not get COVID” group than other two groups (Supplementary Figure S3). These results suggested striking differences in the eating habits of the “Did not get COVID” groups in Italy and Japan.

#### 3.2.3 Herbal medicines, supplements, and essential oils

Herbal medicines have been used for centuries in countries throughout the world. In the questions about the daily usage of herbal medicines, we found that it is rather high in India and Iran (Supplementary Figure S4). “Not tested” group participants in Iran also showed high daily usage of herbal medicine. Daily usage of supplements had the tendency to be higher in the “Not tested” and “Tested positive” groups than the “Did not get COVID” group, and low in the Spanish version (Supplementary Figure S4). Daily usage of essential oil for prevention and recovery was low overall with the tendency to be higher in the “Tested positive” group (Supplementary Figure S4).

### 3.3 Comparison with recovery

**Figure 7:**
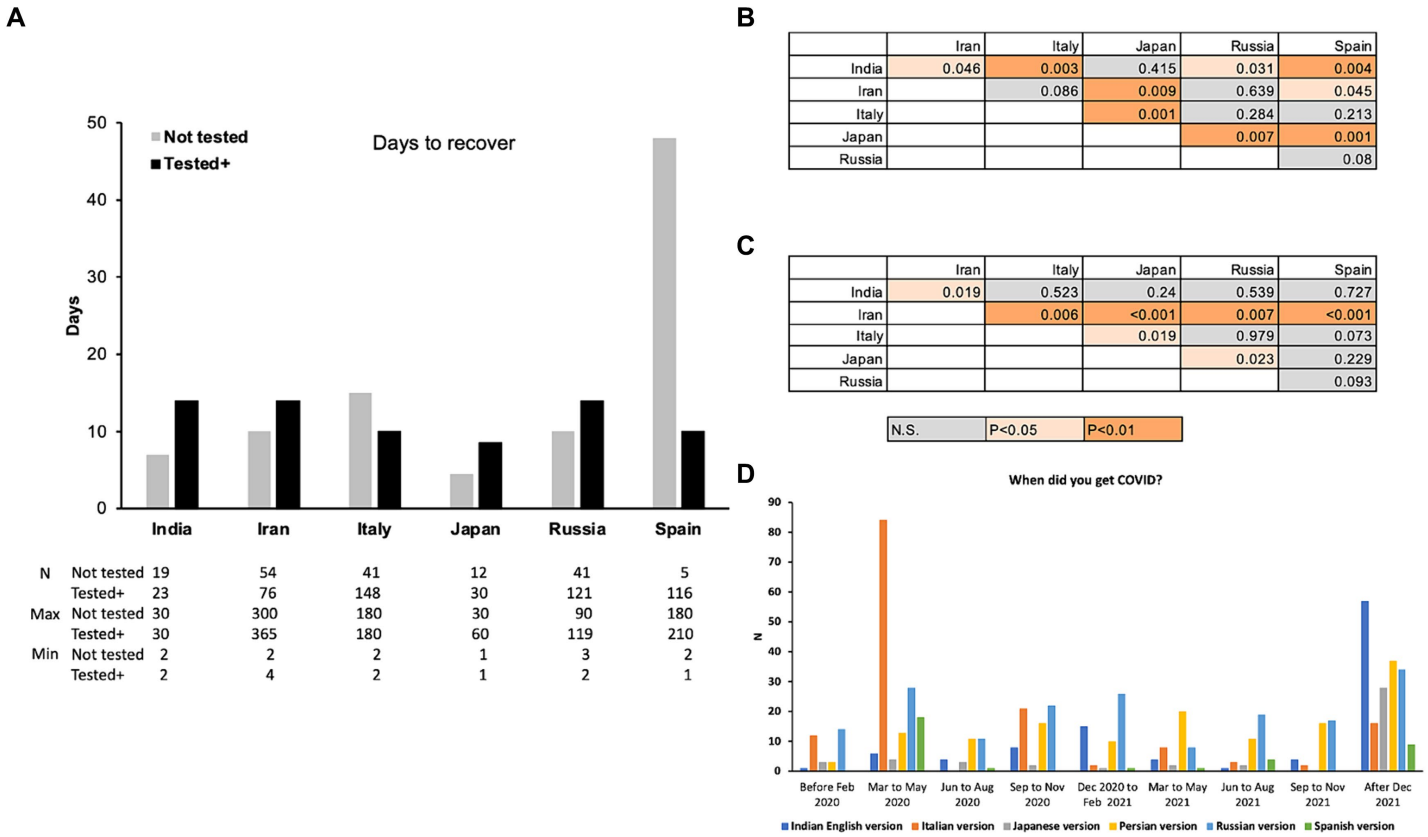
Median of the self-reported time (days) until recovery of “Not tested” and “Tested positive” group participants **(A)** and tables showing statistical differences of pairwise comparisons of the “Not tested” **(B)** and “Tested positive” **(C)** groups. **(D)** Reports on the dates the participants contracted COVID-19 (“Not tested” and “Tested positive” group combined).

There were significant differences in the self-reported days for recovery among the countries. The days for recovery was not necessarily the time until the PCR tests turned negative, but it was the self-reported time length until recovery. The recovery time reported by participants of both “Not tested” and “Tested positive” groups in Japan was statistically significantly shorter than in countries other than India (Figures 7A–C). The recovery time of the “Not tested” group of India was statistically shorter than all other countries except Japan, and the recovery time of the “Tested positive” group of Iran was significantly longer than other countries although the median was not different (Figures 7A–C). The median recovery time of the “Not tested” group of Spain was long, but it was not statistically significantly different from the recovery time of the “Not tested” group of Italy and Russia (Figures 7A,B).

Other than the results of Spain, the median of the number of days until recovery in the “Tested positive” group ranged from 8.5 days (Japan) to 14 days (India, Iran, Russia), and those in the “Not tested” group ranged from 4.5 days (Japan) to 15 days (Italy). The minimum of the reported days ranged from 1 to 4 and the maximum of the reported days ranged from 60 days to 365 (one year), excluding the participants who reported they are still not recovered, most likely with Long-COVID. The days until recovery were significantly longer in the “Tested positive” group than the “Not tested” group in India (Mann–Whitney U-test, U = 122, *p* = 0.014) and Iran (Mann–Whitney U-test, U = 1,108, *p* < 0.001), and not significantly different in other countries. Overall, these results indicate the significant differences among countries and significantly short time to recover in Japan and India, and longer time to recover by the “Tested positive” group in India and Iran.

These differences in the time to recover among countries could be due to the type of the virus (original and variants that emerged later) they contracted and the availability of the medicines especially at the early stage of the pandemic. Figure 7D shows the numbers of answers to the question asking when the participants contracted COVID-19 (“Not tested” and “Tested positive” group combined, and English version excluded as the location of the participants is not clear). Large number of participants in Italy contracted COVID-19 early in 2020 whereas the participants from India, Iran, and Japan show a high peak in “after December” 2021. Spain also showed a high peak in spring 2020 and a smaller peak in “after December 2021.” The number of the participants from Russia shows multiple peaks with slightly higher numbers in spring 2020 and after December 2021.

**Figure 8:**
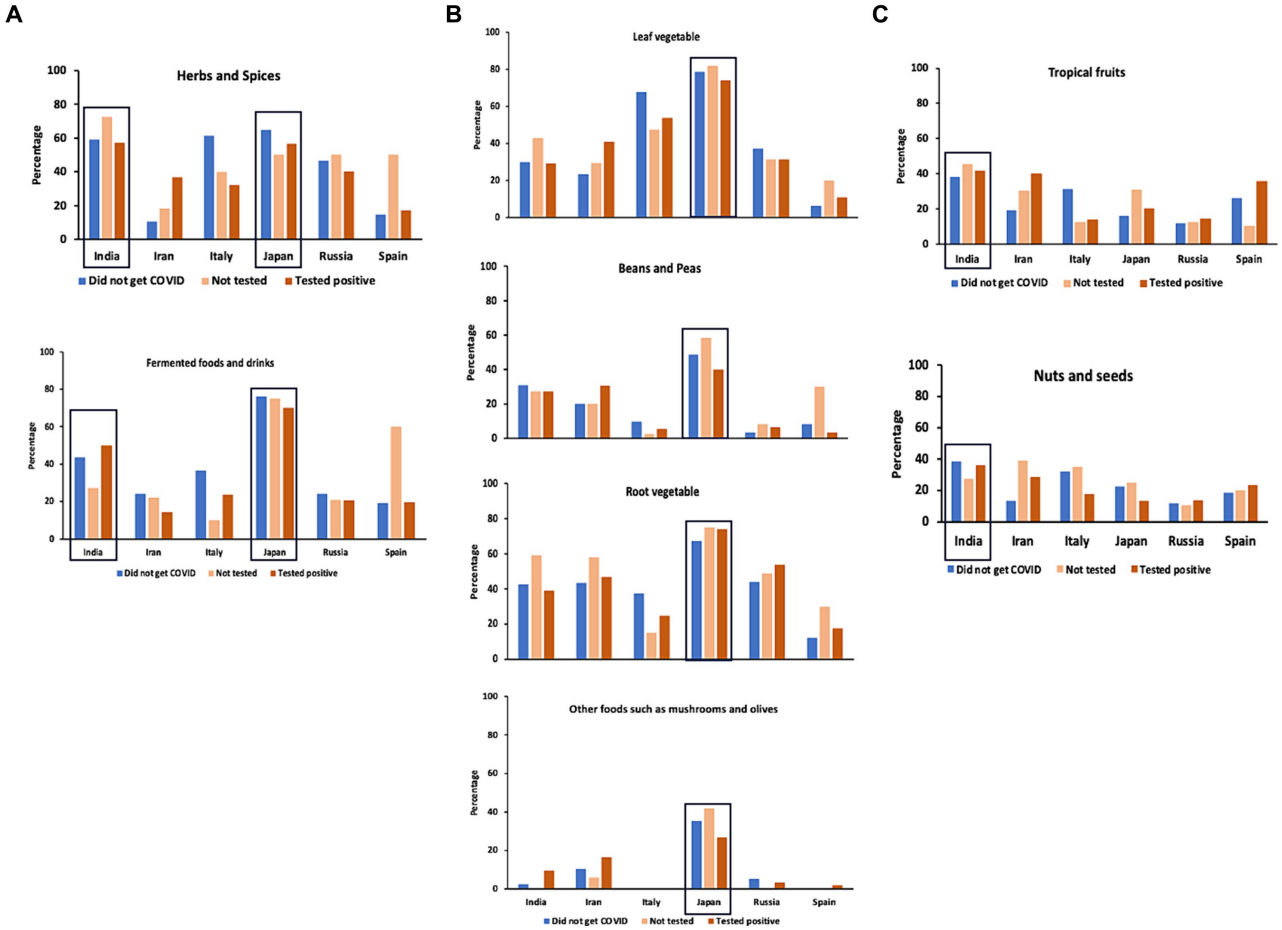
Daily consumption of foods in each country. **(A)** Food categories that showed high percentages of daily consumption in India and Japan. **(B)** Food categories that showed high percentages of daily consumption in Japan than other countries. **(C)** Food categories that showed higher percentages of daily consumption in India than other countries. India and Japan are highlighted by squares.

Although the timing of contracting COVID-19 can affect the time that it may take to recover, there is also a possibility that daily consumption of certain types of foods and beverages can have effects on facilitating recovery from COVID-19. And, if so, there is a possibility that the more people take these foods, the time to recover may become shorter. This could be especially important in case of the “Not tested” group as they were most likely not prescribed medicines, because they were not tested. The food and drink categories that are more frequently consumed in Japan and/or India could suggest the types of foods and beverages that may have such possibly facilitating effects. Figure 8 shows the food that were taken daily and had higher percentages of daily consumption in India and Japan. Beverages did not show specifically higher percentages in Japan and in India than other countries. Hence, they are not shown in Figure 8. Herbs and spices and fermented food/beverages were consumed daily at higher percentages in India and in Japan compared to other countries (Figure 8A). Figure 8B shows the categories of foods that were especially high in Japan and Figure 8C shows the categories of foods that were higher in India than other countries, although the percentages were not extremely high, compared to other food categories. These categories of foods may have had the effects on preventing as well as facilitating recovery from COVID-19 and that these could be the candidate foods for further tests in the future.

Dysfunction in the chemical senses is now well-known as one of the major symptoms of COVID-19. Large number of the participants from Italy replied to have experienced chemosensory dysfunction, and participants from Russia and Spain also replied with a high number of chemosensory dysfunctions. These are the countries where many of the participants reported they contracted COVID-19 early in 2020, when the pandemic was more virulent and the influences of contracting the virus were strongest, and this could be the reason for the higher incidences of chemosensory dysfunction (Supplementary Figure S5).

## 4 Discussion

Our survey suggests that the participants who did not get COVID-19 ate leaf vegetables, root vegetables, other vegetables, and fermented foods/beverages, and used herbs and spices daily at high percentages. In addition, the participants who contracted COVID-19 and answered that they ate vegetables, fermented foods/beverages, and used herbs and spices daily reported fewer days to recover from COVID-19. The key findings from our survey were as follows: (1) Higher percentage of people who did not get COVID-19 daily ate various types of vegetables and fermented foods/beverages. (2) In India and Japan, the people who did not get tested and who tested positive showed significantly shorter time to recover from COVID-19. (3) The people who tested positive or did not get tested and recovered rather quickly were significantly more daily eating/drinking these vegetables and fermented foods/beverages. (4) Fruits are known to have beneficial effects on health, but consumption of fruits was not different between those who contracted and did not contract COVID-19. (5) Teas are also known to have beneficial effects on health and on COVID-19, and people who did not get tested and who did not get COVID-19 were found to have a higher daily intake of tea. (6) People who tested positive reported more severe symptoms compared to people who did not get tested. (7) There were significant differences in the number of days to recover among countries in both the group that did not get tested and the group that tested positive.

Tea and coffee were found to be the most preferred beverages to drink daily, although there were some country-dependent differences about which one is more preferred. In our survey, we found that tea was significantly more consumed daily by the people who did not get COVID-19 and those who were not tested than by the participants who tested positive to COVID-19. Although it is difficult to conclude from survey data whether it had some effects or not, there were significant differences in the severity of the symptoms between people who tested positive and those who did not get tested. Previous studies have shown that (−) epigallocatechin gallate (EGCG), theasinensin A (TSA), and galloylated theaflavins such as theaflavin 3,3′-di-O-gallate (TFDG) included in teas have anti-SARS-CoV-2 activities (13, 14, 24, 45, 46). The differences in the processing methods generate large differences in the concentration of chemical composition in green tea, yellow tea, white tea, oolong tea, and black tea (47). TSA and TFDG are catechin derivatives that increase by enzymatic oxidation during the processing of the tea leaves, and thus EGCG is decreased in black tea (47, 48). The studies showing that EGCG, TSA, and TFDG have anti-SARS-CoV-2 effects indicate that these changes in the amount included by differences in the processing methods do not affect the potential positive effects of tea. Importantly, it should be noted that there are studies showing that milk casein blocks the anti-SARS-CoV-2 effects of tea because of the binding of them to casein (49). These studies suggest the possible effects of teas on preventing and suppressing severity of COVID-19, but also indicate that depending on how the tea is prepared (for example, milk tea), the effects can be blocked.

Although in our survey the results on coffee did not show its effects on facilitating recovery from COVID-19 or suppressing infection, a study using a large amount of data (n > 37 K) in the UK Biobank on dietary habits and COVID-19 infection rate has shown a significant negative correlation between the drinking habit of coffee and the infection rate, i.e., people drinking coffee were less infected to COVID-19 (50). An *in vitro* study has also shown that coffee, caffeine, or diluted human serum samples collected from healthy adults who drank coffee suppressed infection of SARS-CoV-2 (25). It could be that the lack of tendency in our project is due to the smaller data size, and it is also possible that the differences are due to the international nature of our study, or possible interactions with other beverages or foods or the way of preparation like in case of teas.

An interesting result in this study is that the people who did not get COVID-19 showed general trend to consume leaf, root and other vegetables, mushrooms, fermented foods/beverages, beans and peas, and herbs and spices daily and that the people who tested positive and those who did not get tested and consumed them daily recovered faster. This tendency of inverse correlation between intake of vegetables and COVID-19 was also found in previous COVID-19 literature. Tadbir Vajargah et al. (51) found that the severity of COVID-19 symptoms of 250 hospitalized patients negatively correlated with the amount of consumption of vegetables as well as fruits and dietary fiber (51). They also recovered faster, leading to shorter hospitalization and convalescence periods. A meta-analysis study, which was conducted before the COVID-19-pandemic, has also shown that higher intakes of fruits and vegetables reduce proinflammatory cytokines, and improved the immune system profiles (52). In a survey-based study focusing on six European countries, researchers found that people who were on plant-based diet or pescatarian diet (plant-based diet with fish) had milder symptoms of COVID-19 (16). The results of our study are in line with these studies and suggest the importance of vegetable intake and also the importance of studies on the key phytochemicals or combinations of phytochemicals that contributed to these differences.

In the case of fermented foods and beverages, there are additional factors that are necessary to take into consideration, i.e., the roles of bacteria. There are also animal-based fermented foods/beverages, which may have different effects compared to those that are plant-based. Muhialdin et al. (40) summarized the antiviral activities of probiotic bacteria in fermented foods. They suggested that the antiviral effects of fermented foods could be exerted directly through the viral neutralization by the bioactive compounds and indirectly through improving the innate immune system (40). It should be noted that the fermented foods/beverages category was one of the two most commonly reported categories in India and Japan; the two countries that showed significantly faster recovery from COVID-19. This suggests the importance of further studies on fermented foods/beverages.

Considering the well-known beneficial effects of fruits on health, we hypothesized that we would find higher daily consumption of fruits, but, interestingly, with the exception of stone fruits, daily fruit consumption was rather low compared to vegetables and fermented foods. In fact, there are studies that found the negative association of fruits consumption and COVID-19 (51). One study identified foods that contain chemical compounds with bioactive properties that resemble clinically approved drugs for COVID-19, and they identified 52 phytochemical compounds with anti-SARS-CoV-2 effects (21). They reported that flavonoids, coumarins, stilbenes, indoles, and phenolic acids are the major groups of phytochemicals with anti-COVID-19 effects, and examples of the phytochemicals in these groups are quercetin, kaempferol, myricetin of flavonols, luteolin and apigenin of flavones, procyanidin B2 of flavanols, naringin of flavanones, daidzein, genistein, legumelin of isoflavonoids, trans-resveratrol of stilbenes, 3-indole-carbinol of indoles, and gallic acid of phenolic acids. Many of these phytochemicals with bioactive properties against SARS-CoV-2 are present in fruits (berries, citrus fruits, pome fruits, stone fruits, and tropical fruits). It could be that, similarly to coffee, there may be some differences in the images of fruits among countries (for example, fruits are called “mizu-gashi” in old Japanese language, which means “watery sweets/desserts”). Japanese participants reported very high daily intake of all types of vegetables and fermented foods/drinks in comparison to other countries, but daily consumption of fruits was rather low. Daily consumption of citrus fruits and tropical fruits was high in India and Iran, and Iran showed higher daily consumption of pome fruits than other countries. These differences could be an example of cultural differences in eating habits among countries.

We were unable to control individual differences in the amount of each serving size of food and beverages. There are also differences in the amount of chemical ingredients even within the same plant, depending on its location and seasonal differences, and in the way the food/beverage is prepared. The nutrients also differed depending on whether they were cooked or raw. Another issue to take into consideration is the possibility that the results of this survey could be telling that the people who consume various vegetables daily are more cautious of their overall health and may modify other activities of daily living other than their eating habits, such as self-distancing, wearing masks, and sanitizing their hands and other things. As such, there are also limits in the study using surveys.

In conclusion, our results suggest that daily consumption of phytochemical compounds included in the vegetables may have contributed to preventing the onset of COVID-19 as well as to accelerating the recovery from COVID-19. The food and beverage categories that were found to be eaten daily more by the people who did not get COVID-19 are the suitable candidates to conduct studies in detail with more controlled experimental conditions, studies using animal model systems, and at the level of phytochemical compounds to determine mechanisms of action in future.

## Data availability statement

The raw data supporting the conclusions of this article will be made available by the authors, without undue reservation.

## Ethics statement

The survey contained a question that asked consent to participate and we obtained consent from the participants. Ethical approval was obtained as an exempt study from Indiana University Human Research Protection Program (HRPP) in the U.S.A. (protocol #14915), the Office of Regulatory Research Compliance of Howard University (IRB-2022-0380), the Jikei University School of Medicine Ethics Committee in Japan (#34–003), the Bioethics Committee at the A.N. Severtsov Institute of Ecology & Evolution of Russian Academy of Sciences (no.2022-63-NC), and by the Second Affiliated Hospital of Xi’an Jiaotong University, Medical Ethics Committee in China (#2022023). The studies were conducted in accordance with the local legislation and institutional requirements. The participants provided their written informed consent to participate in this study.

## Author contributions

SK: Writing – review & editing, Writing – original draft, Visualization, Supervision, Resources, Project administration, Methodology, Formal analysis, Data curation, Conceptualization. PJ: Investigation, Funding acquisition, Writing – review & editing, Supervision, Project administration, Conceptualization. VS: Visualization, Validation, Writing – review & editing, Supervision, Project administration, Conceptualization. TH: Methodology, Writing – review & editing, Supervision, Project administration, Conceptualization. PA: Writing – review & editing, Formal analysis, Data curation. RKa: Writing – review & editing, Validation, Supervision, Project administration, Methodology, Investigation, Formal analysis, Data curation. RKu: Writing – review & editing, Project administration, Investigation, Formal analysis, Data curation, Conceptualization. RA: Writing – review & editing, Supervision, Methodology, Investigation, Formal analysis. SB: Writing – review & editing, Methodology, Investigation, Conceptualization. OC: Writing – review & editing, Methodology, Investigation. CM-C: Writing – review & editing, Project administration, Methodology, Investigation, Data curation. JC: Writing – review & editing, Methodology, Investigation. KC: Writing – review & editing, Formal analysis, Data curation. SD: Writing – review & editing, Validation, Supervision, Methodology, Investigation. PR: Writing – review & editing, Project administration, Methodology, Investigation. MG: Writing – review & editing, Methodology, Investigation, Data curation. MK: Writing – review & editing, Methodology, Investigation, Formal analysis, Data curation. TL: Writing – review & editing, Methodology, Investigation, Data curation. EM: Writing – review & editing, Methodology, Investigation. ZN: Writing – review & editing, Methodology, Investigation, Data curation. HN: Writing – review & editing, Methodology, Investigation, Formal analysis, Data curation. MÖ: Writing – review & editing, Investigation. SP: Writing – review & editing, Methodology, Investigation. EÖ: Writing – review & editing, Methodology, Investigation. DS: Writing – review & editing, Methodology, Investigation. FT-H: Writing – review & editing, Methodology, Investigation. RU: Writing – review & editing, Methodology, Investigation. VV: Writing – review & editing, Project administration, Methodology, Investigation, data curation.

## Footnotes

^1^ https://gcchemosensr.org/

^2^ https://www.qualtrics.com
