# Supplementary material for: Possible roles of phytochemicals with bioactive properties in the prevention of and recovery from COVID-19
Source: Front Nutr. 2024 Jul 10;11:1408248. doi: 10.3389/fnut.2024.1408248 (PMC11266003; doi:10.3389/fnut.2024.1408248)
Supplement: Supplementary file 1 [file Data_Sheet_1.docx]

Supplementary Material

Possible roles of phytochemicals with bioactive properties in the prevention of and recovery from COVID-19

**Sachiko Koyama*, Paule V. Joseph*, Vonnie D.C. Shields, Thomas Heinbockel, Poonam Adhikari, Rishemjit Kaur, Ritesh Kumar, Rafieh Alizadeh, Surabhi Bhutani, Orietta Calcinoni, Carla Mucignat-Caretta, Jingguo Chen, Keiland W. Cooper, Subha R. Das, Paloma Rohlfs Domínguez, Maria Dolors Guàrdia, Maria A. Klyuchnikova, Tatiana K. Laktionova, Eri Mori, Zeinab Namjoo, Ha Nguyen, Mehmet Hakan Özdener, Shima Parsa, Elif Özdener Poyraz, Daniel Jan Strub, Farzad Taghizadeh-Hesary, Rumi Ueha, Vera V. Voznessenskaya**

*** Correspondence:** Sachiko Koyama: [sakoyama@iu.edu](mailto:sakoyama@iu.edu), Paule V. Joseph: [paule.joseph@nih.gov](mailto:paule.joseph@nih.gov)

# Supplementary Methods

# Development of Survey

# Questions about eating habits were constructed by asking how frequently the participants ate each category of foods, and the category was based on the food classification by the Ministry of Health Labour and Welfare of Japan (https://www.mhlw.go.jp/english/topics/foodsafety/positivelist060228/dl/r04.pdf). The food categories that we used were: citrus fruits, berries, pome fruits, stone fruits, tropical fruits, other fruits, leaf vegetables, root vegetables, other vegetables, beans and peas, nuts and seeds, herbs and spices, cereals and grains containing buckwheat and barleys, other foods such as mushrooms, and fermented foods. Buckwheat and barleys were specifically selected as representatives of cereals and grains and based on a paper showing that the phytochemicals included in them were effective on COVID-19 (Laponogov *et al.*, 2021). In order to enhance the understanding of the types of foods and the ease of answering survey questions, we listed some examples of foods in the questions pertaining to food categories. For example, in asking the frequency of eating root vegetables, we asked “Root vegetables, such as carrot, turnip, garlic, beetroots, parsnip roots, parsley root, radish, onion, sacred lotus, taro, yam, sweet potato, potato?” (English version). As the participants took the survey from around the world, it was necessary to adjust the examples depending on the language version because of the differences in the availability and familiarity of certain food items. In addition, we did not want the availability of the examples to affect the answers. There were also some food items that were named differently, depending on their location. Thus, the Indian English version was prepared separately. Questions on drinking habits were constructed by asking how frequently the participants drank four types of beverages: 1) Tea, based on the studies showing the chemical ingredients found within, had effects on coronavirus (Chourasia *et al.*, 2021; Ohgitani *et al.*, 2021), 2) other tea and herbal teas, not made from tea plants, 3) coffee, 4) cider, 5) hot chocolate, and 4) alcohol. Similar to the questions pertaining to foods, we showed some examples to enhance the ease of understanding. In addition, we made some adjustments to the examples shown in each language version of the survey (for example, the Russian and Turkish versions had examples of vodka and raki, respectively, instead of brandy, which other language versions may have contained). The Persian version did not have questions on cider and alcohol. The lists of these examples are shown in Supplementary Table 1.

# Regarding COVID-19, we asked to choose from the choices of “Did not get COVID”, “Not get tested but think yes”, and “Tested positive”. We also asked if they got COVID-19, when they got sick and how long it took to recover (not necessarily based on PCR results as there was the “Not tested” group). We asked what symptoms they experienced as well as their severity. The symptoms that were asked included if people experienced a headache, cough, shortness of breath, runny nose or congestion, muscle/body aches/soreness, chills, lightheadedness or brain fog, dizziness, nausea, fatigue, memory loss or concussion or hallucination, diarrhea or gastrointestinal symptoms, hair loss, smell loss or reduction, taste loss or reduction, chemesthesis loss or reduction, other symptoms. Separately, we asked if they have persisting symptoms (PASC, or Long-COVID).

# The English version of the questionnaire was developed first using Qualtrics software (Qualtrics, Provo, UT, USA, https://www.qualtrics.com). Subsequently, the survey was translated into nine other languages, thus getting a total of 10 language versions, namely Chinese, English, Indian English, Italian, Japanese, Persian, Polish, Russian, Spanish, and Turkish. The survey has been online since February 2022. Data used for the analysis were obtained between February 2022 and May 2023. The frequency of eating and drinking was asked following the style of the Diet History Questionnaire (DHQ) by the National Cancer Institute (NCI) (Subar *et al.*, 2001) as a validated questionnaire for surveys on nutrition and modified the choices of answers of DHQ to “Never”, “Daily”, “Weekly”, “Monthly”. We also asked the people surveyed which type of food items and beverages they consumed using a multiple selection question. As the examples do not cover all the types of foods in each category, we also added a question where survey participants could include what food or beverage item they ate or drank.

References

Chourasia, M. *et al.* (2021) ‘EGCG, a Green Tea Catechin, as a Potential Therapeutic Agent for Symptomatic and Asymptomatic SARS-CoV-2 Infection’, *Molecules*, 26(5), p. 1200. Available at: https://doi.org/10.3390/molecules26051200.

Laponogov, I. *et al.* (2021) ‘Network machine learning maps phytochemically rich “Hyperfoods” to fight COVID-19’, *Human Genomics*, 15(1), p. 1. Available at: https://doi.org/10.1186/s40246-020-00297-x.

Ohgitani, E. *et al.* (2021) ‘Significant Inactivation of SARS-CoV-2 In Vitro by a Green Tea Catechin, a Catechin-Derivative, and Black Tea Galloylated Theaflavins’, *Molecules*, 26(12), p. 3572. Available at: https://doi.org/10.3390/molecules26123572.

Subar, A.F. *et al.* (2001) ‘Comparative Validation of the Block, Willett, and National Cancer Institute Food Frequency Questionnaires’, *American Journal of Epidemiology*, 154(12), pp. 1089–1099. Available at: https://doi.org/10.1093/aje/154.12.1089.

# Supplementary Figures and Tables

For more information on Supplementary Material and for details on the different file types accepted, please see [here](https://www.frontiersin.org/guidelines/author-guidelines#supplementary-material).

## Supplementary Figures


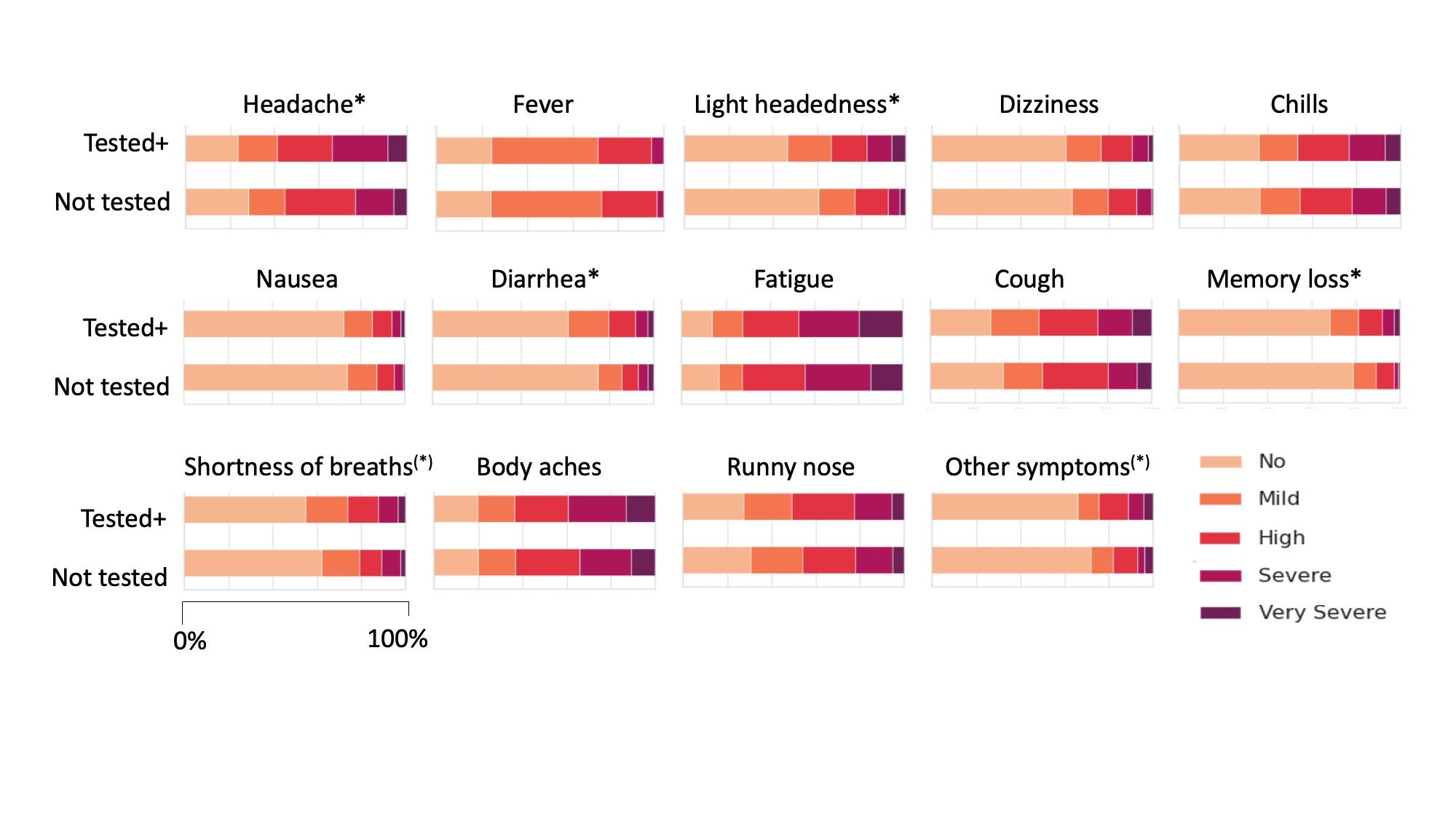


**Supplementary Figure 1.** Symptoms of COVID-19 reported by the “Tested Positive” group and “Not Tested” group. *: P<0.05, (*): 0.05<P<0.10


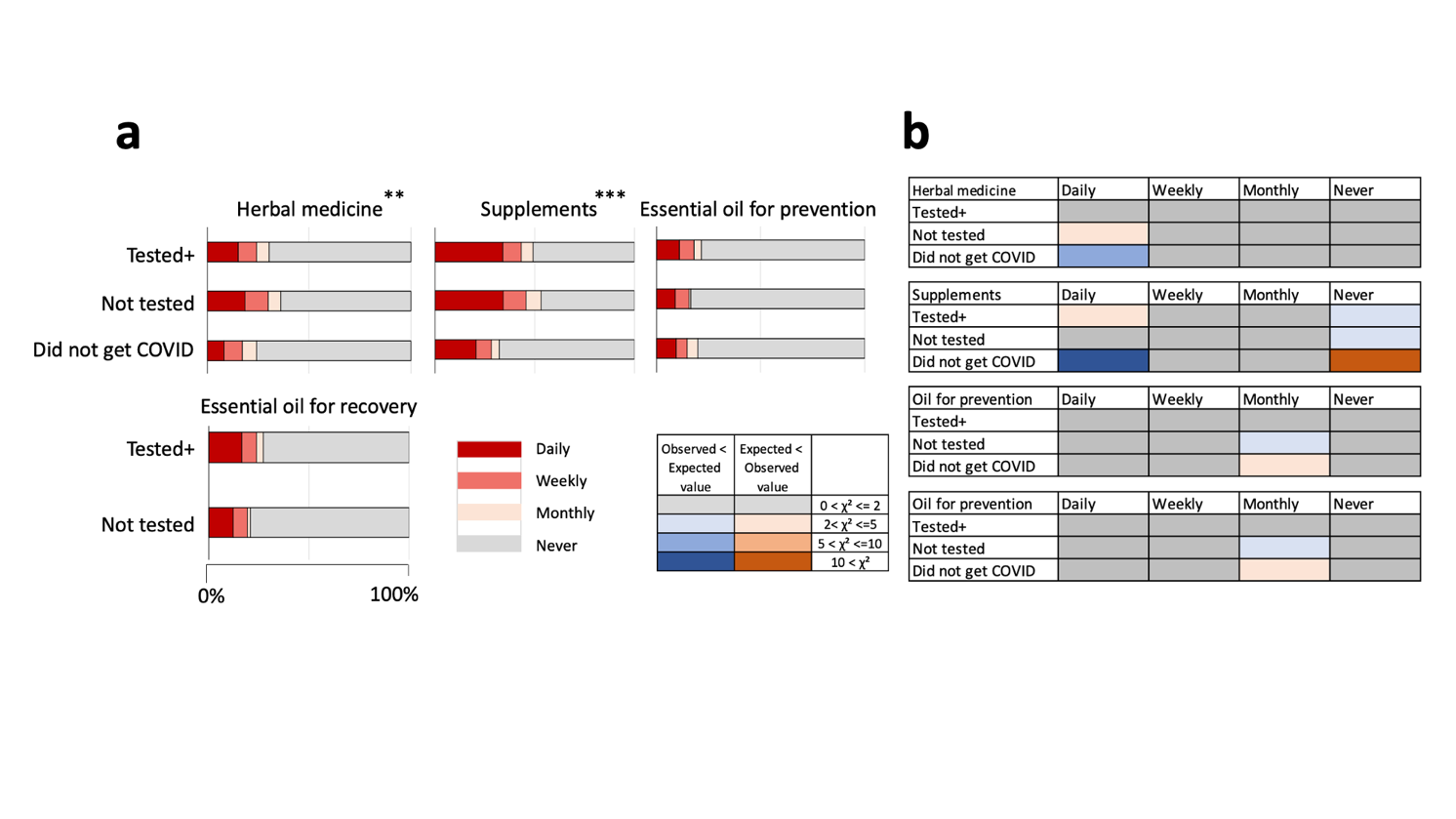


**Supplementary Figure 2.** Intake of herbal medicines, supplements, and usage of essential oils. (a) Comparison of percentages of fruits intake. Chi-square test, herbal medicine, χ²=18.872, df=6, P=0.004; supplements, χ²=38.246, df=6, P<0.001; essential oils for prevention, χ²=10.904, df=6, N.S.; essential oils for recovery, χ²=4.807, df=3, N.S. (b) Discrepancy from expected values. The colors indicate where the observed values were higher than the expected values (smaller to larger discrepancy indicated by pink to red color), and the observed values were lower than the expected values (smaller to larger discrepancy indicated by light to dark blue). Grey color indicates there were no or negligible discrepancies: Results on herbal medicines, supplements, essential oils.


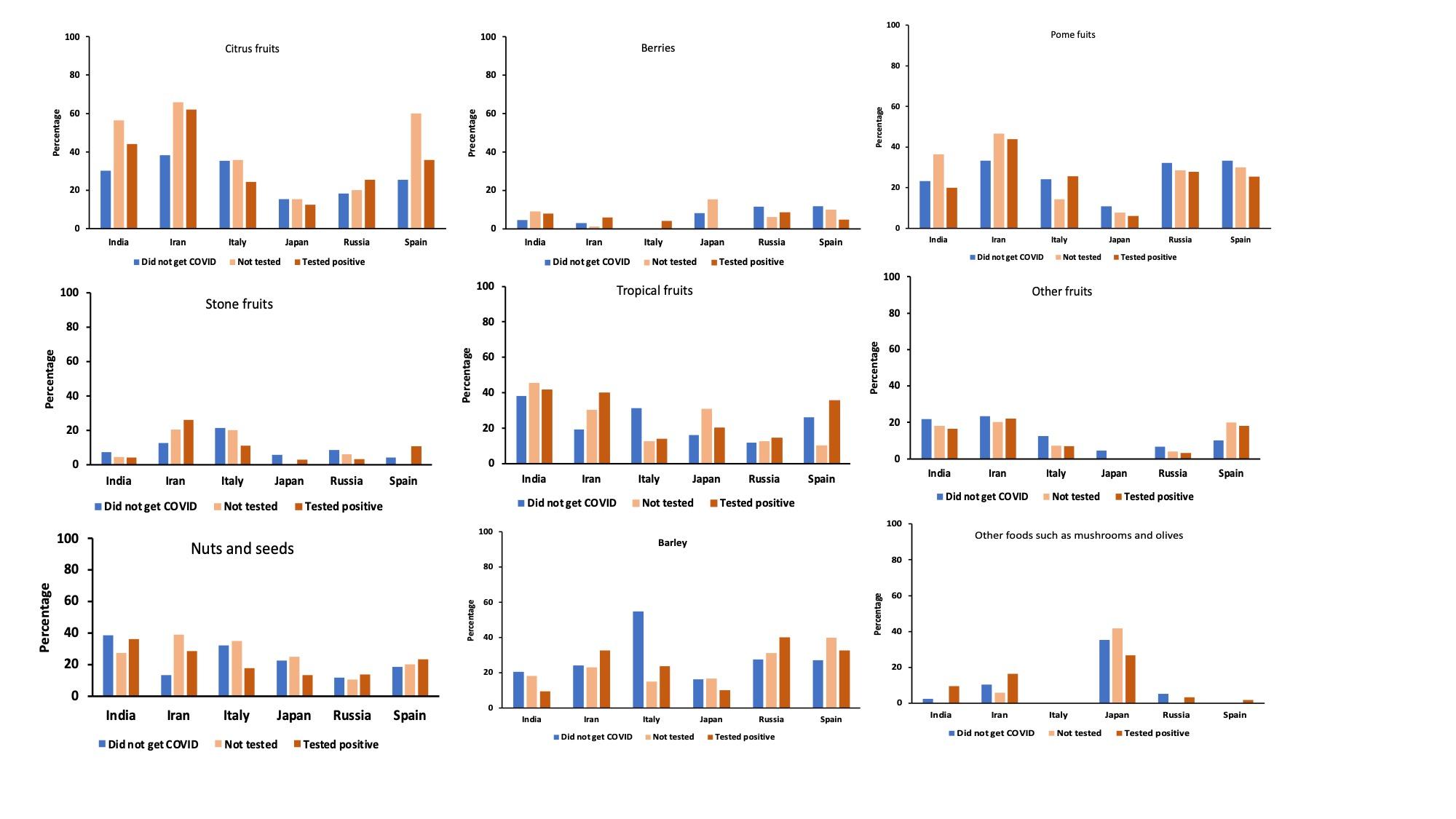


**Supplementary Figure 3.** Daily intake of foods of categories other than those shown in the main text, split by countries. Blue bars: “Did not get COVID-19” group, light orange bars: “Not tested” group, Dark orange bars: “Tested positive” group.


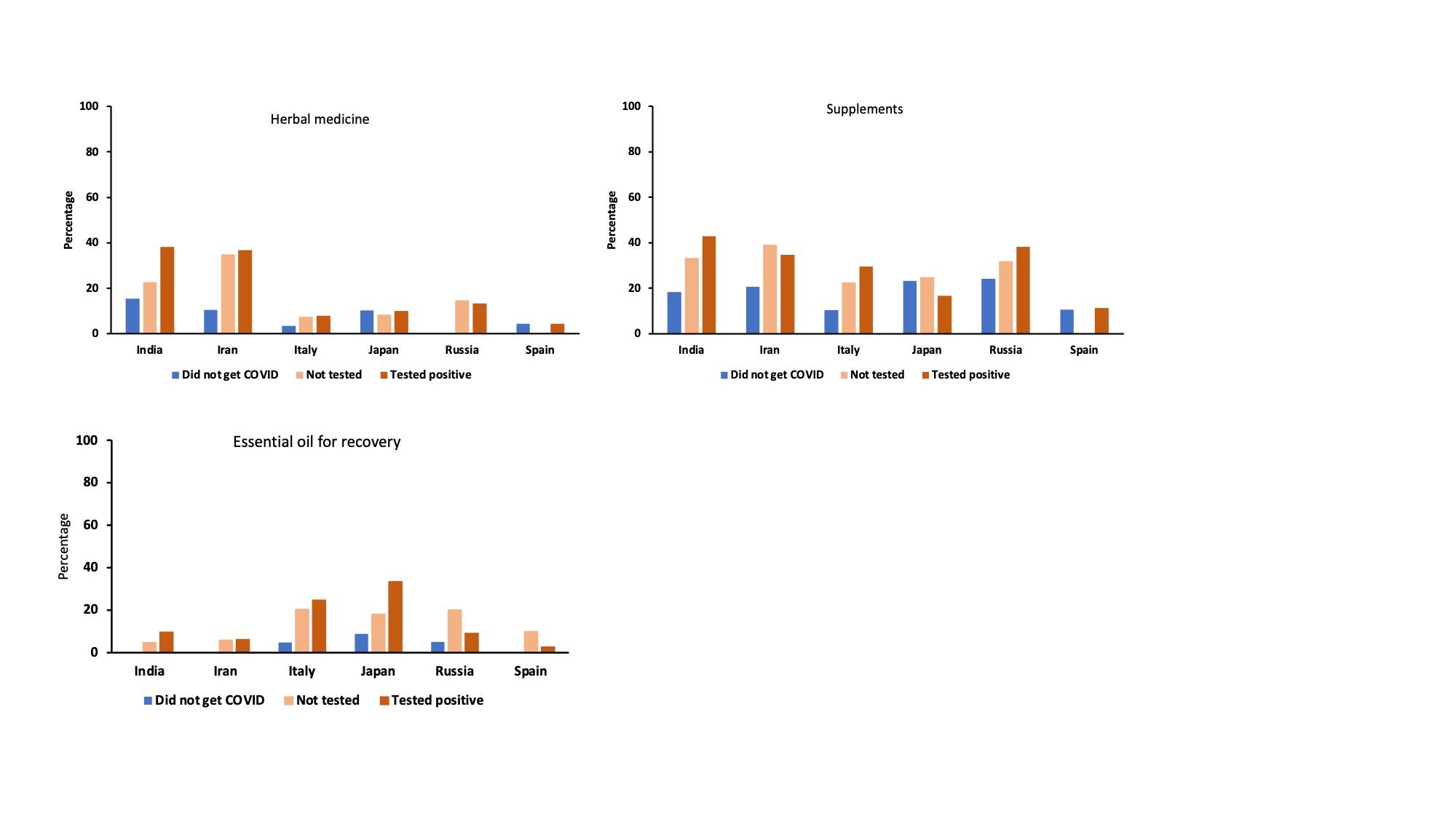


**Supplementary Figure 4.** Daily intake of herbal medicine and supplements, and daily use of essential oils, split by countries.


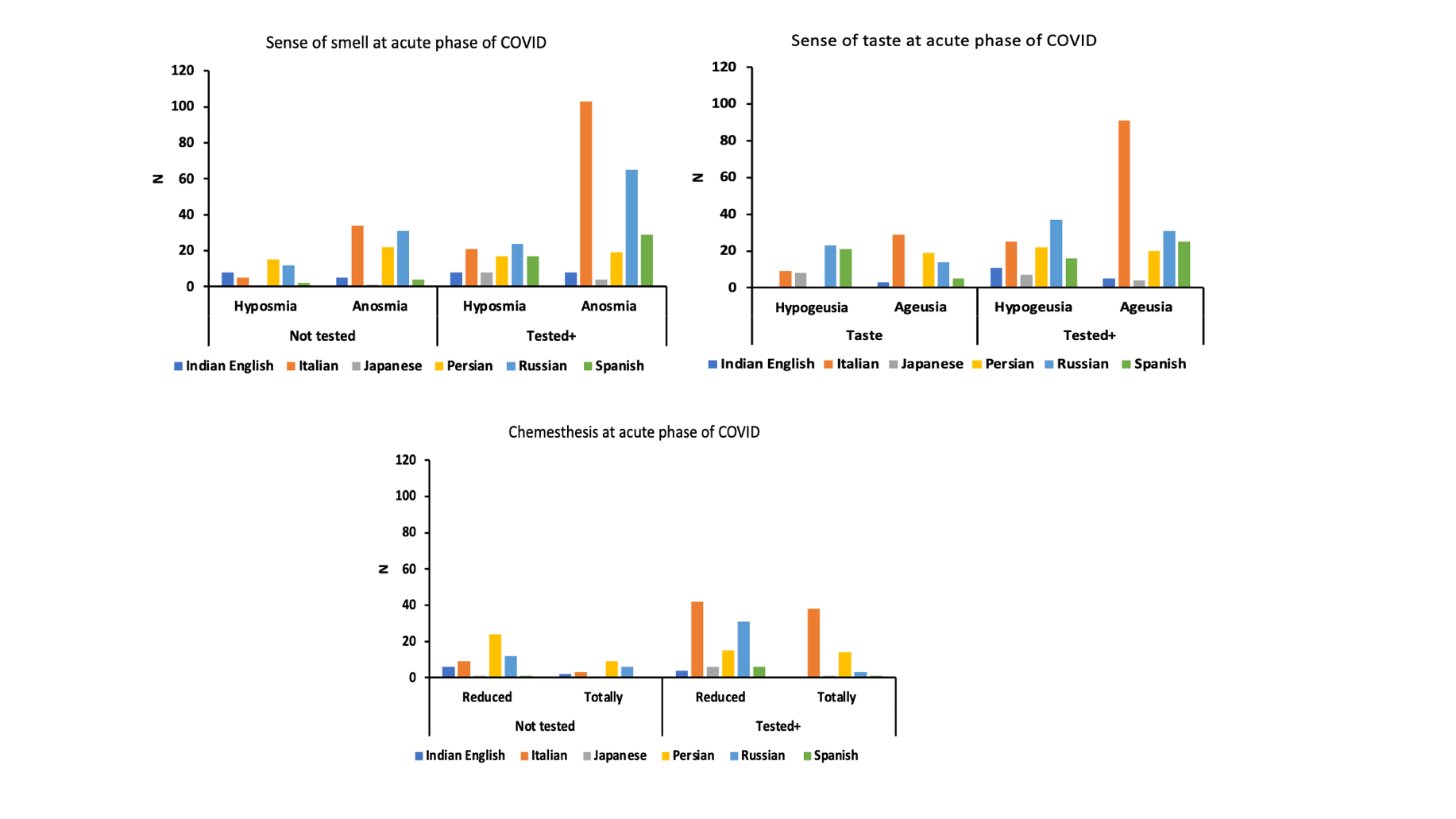


**Supplementary Figure 5.** Number of participants reporting experiences of chemosensory dysfunction due to COVID-19.

**2.2. Supplementary Tables**

**Supplementary table 1**. Examples of food items used in each language version of the survey (Part 1: Chinese, English, Indian English, and Japanese)

|  | Chinese | English | Indian English | Italian | Japanese |
| --- | --- | --- | --- | --- | --- |
| Tea | 红茶、绿茶、白茶、黄茶、乌龙茶  (Translation into English: black tea, green tea, white tea, yellow tea, oolong tea) | black tea, green tea, white tea, yellow tea, oolong tea, red tea | black tea, green tea, red tea | tè nero, tè verde, tè bianco, tè giallo, tè oolong, tè rosso  (Translation into English: black tea,  green tea, white tea, yellow tea, oolong tea,  red tea | 紅茶、緑茶、烏龍茶、  チャイティー、麦茶  (Translation into English: black tea,  green tea, oolong tea, chai tea, wheat tea) |
| Herbal tea | 姜茶、柠檬草茶、甘草茶(Translation into English: ginger tea, lemongrass tea, licorice tea) | Ginger tea, lemongrass tea, licorice tea | Ginger tea, lemongrass tea, mulethi (liquorice) tea, tulsi tea, cinnamon tea | zenzero, citronella, liquirizia  (Translation into English: ginger tea, lemongrass tea, licorice tea) | 生姜茶、レモングラスティー、甘草茶  (Translation into English: ginger tea, lemongrass tea, licorice tea) |
| Coffee | 咖啡 (Translation into English: coffee) | Coffee | Coffee | Caffè (Translation into English: coffee) | コーヒー (Translation into English: coffee) |
| Hot chocolate | 可可 (Translation into English: cocoa) | Cocoa | hot chocolate | Cioccolata (Translation into English: chocolate) | ココア(Translation into English: cocoa) |
| Cider | 碳酸饮料  (Translation into English: cider) | Cider | Apple cider | Sidro  (Translation into English: cider) | 炭酸飲料 (Translation into English: cider) |
| Alcohol | 葡萄酒、啤酒 (Translation into English: wine, beer, whiskey, brandy) | wine, beer, whiskey, brandy, others | wine, beer, whiskey, brandy, others | vino, birra, whiskey,  brandy (Translation into English: wine, beer, whiskey, brandy) | ワイン、ビール、  ウイスキー、ブランデー (Translation into English: wine, beer, whiskey, brandy) |
| Citrus fruits | 橙子、桔子、柠檬、酸橙、柚子、金桔、甜橙(Translation into English: Oranges, tangerines, lemons, limes, grapefruits, kumquats, sweet oranges) | mandarin orange, clementine, tangerine, lemon, lime, pummelo, kumquat, sweet orange | kinnow, lemon, lime, mausami, orange | mandarino, arancia,  limone, lime, clementine,  kumquat, pomelo, mandarancio  (Translation into English: tangerine,  orange, lemon, lime, clementine, kumquat,  pomelo, mandarancio) | みかん、 オレンジ、ぶんたん、レモン、ライム、ゆず、すだち、キンカン、グレープフルーツ (Translation into English: mandarin orange, orange, buntan, lemon,  lime, yuzu, sudachi,  kumquat, grapefruit) |
| Berries | 蔓越莓、蓝莓、越橘、草莓、黑莓、覆盆子、蔓越莓、黑接骨木、醋栗、黑桑葚、巴西针叶樱桃、黑加仑（黑加仑甜酒）(Translation into English: Cranberries, blueberries, lingonberries, strawberries, blackberries, raspberries, cranberries, black elderberries, gooseberries, black mulberries, acerola, blackcurrants (blackcurrant liqueur)) | cranberry, blueberry, huckleberry, strawberry, blackberry, raspberry, black elderberry, gooseberry, black mulberry, pitanga, black currants (cassis) | blueberry, strawberry, jamun (blackberry), raspberry, cranberry, amla (gooseberry) | mirtillo rosso, fragola,  mora, mora di gelso,  ribes nero (cassis),  sambuco,  mirtillo selvatico, uva spina, mirtillo coltivato,  pitanga (ciliegia di cayenna), lampone  (Translation into English: cranberry, strawberry, blackberry, mulberry, cassis, elderberry, wild blueberry, gooseberry, cultivated blueberry, pitanga (cayenne cherry), raspberry) | イチゴ、ブルーベリー、カシス、ラズベリー、クランベリー、ブラックベリー、スグリの実（グースベリー）(Translation into English: strawberry, blueberry,  cassis, raspberry, cranberry, blackberries,  gooseberry) |
| Pome fruits | 苹果、梨、榅桲 (Translation into English: apples, pears, quinces) | apple, pear, quince | apple, pear | mela, pera, cotogna  (Translation into English: apple, pear,  quince) | リンゴ、梨、洋梨、  カリン、柿、びわ (Translation into English: apple  Asian pear, pear, karin,  persimmon, loquat) |
| Stone fruits | 李子、樱桃、杏、桃子（含加工后的食品）(Translation into English: plums, cherries, apricots, peaches (including processed foods)) | plum, cherry, apricot, peach | plum, cherry, apricot, peach | prugna, ciliegia, albicocca, pesca  (Translation into English: plum, cherry, apricot, peach) | 桃、さくらんぼ、スモモ、プラム、梅 (Translation into English: peach,  cherry, sumomo, plum, ume) |
| Tropical fruits | 香蕉、芒果、木瓜、猕猴桃、菠萝、椰枣、牛油果、刺果、番荔 (Translation into English: Banana, mango, papaya, kiwi, pineapple, date, avocado, thorn fruit, cherimoya) | banana, mango, papaya, kiwi, pineapple, date, avocado, soursop | banana, mango, papaya, kiwi, pineapple, date, avocado | banana, mango, papaya,  ananas, datteri, avocado,  kiwi, guanabana  (Translation into English: banana,  mango, papaya, pineapple, dates, avocado, kiwi, soursop) | バナナ、マンゴー、パパイヤ、キウイ、パイナップル、アボカド、ナツメヤシ (Translation into English: banana, mango, papaya, kiwi, pineapple, avocado, dates) |
| Other fruits | 无花果、甜瓜、西瓜、葡萄  (Translation into English: figs, melons, watermelons, grapes) | fig, muskmelon, watermelon, and grapes | fig, muskmelon, watermelon, and grapes | fichi, melone, anguria, uva  (Translation into English: figs, melon,  watermelon, grape) | イチジク、メロン、スイカ、ブドウ、ザクロ (Translation into English: fig, melon,  watermelon, grapes,  pomegranate) |
| Leaf vegetables | 生菜、菊苣、莴苣菜、萝卜叶、菠菜、芹菜、马齿苋、卷心菜、西兰花、甘蓝小包菜、花椰菜、甜菜叶、茴香、芝麻菜、洋葱的绿色部分（叶）  (Translation into English: Lettuce, endive, endive, turnip greens, spinach, celery, purslane, cabbage, broccoli, kale, cauliflower, beet greens, fennel, arugula, green part (leaves) of onions) | lettuce, chicory, endive, turnip leaves, spinach, celery, purslane, cabbage, broccoli, brussel sprouts, cauliflower, beet leaves, fennel, arugula, green parts (leaves) of onions, and artichokes | lettuce, leaves of turnip, leaves of mustard, spinach, celery, cabbage, broccoli, brussel sprouts, cauliflower, beet leaves, fennel, spring onions | lattuga, cicoria, indivia,  foglie (cime) di rapa,  spinaci, sedano, portulaca, cavoli, broccoli, cavolini di Bruxelles, cavolfiori,  biete, finocchio, rucola,  parti verdi (foglie) di cipolla, Carciofi  (Translation into English: lettuce,  chicory, endive, turnip leaves (greens), spinach,  celery, purslane, cabbage,  broccoli, Brussels sprouts, cauliflower, beets, fennel, rocket, green parts (leaves) of onion, artichokes) | レタス、キャベツ、芽キャベツ、ブロッコリー、カリフラワー、セロリ、ほうれん草、カブやダイコンの葉の部分、青ネギ・ネギ・タマネギの緑の部分、チコリ、エンダイブ、ツルムラサキ、ビートの葉、チャイニーズマスタード、フェンネル、  ルッコラ、アーティチョーク (Translation into English: lettuce, cabbage,  Brussels sprouts, broccoli, cauliflower,  celery, spinach, the leaf parts of turnips and radish, green onions, green onions, green parts of onions,  chicory, Endive, tsurumurasaki,  beet leaves, Chinese mustard, fennel, arugula, artichoke) |
| Root vegetables | 胡萝卜，朝鲜蓟，萝卜，姜黄，大蒜，姜，甜菜根，欧防风根，欧芹根，小红萝卜，洋葱根，莲，芋头，山药，甘薯，土豆(Translation into English: Carrots, artichokes, radish, turmeric, garlic, ginger, beetroot, parsnip root, parsley root, radish, onion root, lotus root, taro, yam, sweet potato, potato) | carrot, turnip, garlic, beetroots, parsnip roots, parsley root, radish, onion, sacred lotus, taro, yam, sweet potato, potato | carrot, turnip, garlic, beetroots, radish, onion roots, sacred lotus, arbi (taro), sweet potato, potato | carota, rapa, aglio, barbabietola, radici di pastinaca, radici di prezzemolo, ravanello,  cipolla, loto, taro, igname, patata dolce,  patata  (Translation into English: carrot,  turnip, garlic, beet,  parsnip roots, parsley roots, radish, onion,  lotus, taro, yam, sweet potato, potato) | ダイコン、ゴボウ、ニンジン、タマネギ、レンコン、サツマイモ、ジャガイモ、タロイモ、里芋、山芋（とろろ）、ヤムイモ、ニンニク、カブ、ラディッシュ、根パセリ、パースニップ、ビートルート (Translation into English: lotus root, sweet potato, potato, taroimo, satoimo, yamaimo (Tororo), yam, garlic, turnip, radish, parsley root, parsnip, beetroot) |
| Other vegetables | 黄瓜、葫芦、芦笋、韭菜、小番茄、圣女果、青椒、意大利甜红椒、橙椒、黄椒、茄子、秋葵 (Translation into English: cucumbers, gourds, asparagus, leeks, cherry tomatoes, cherry tomatoes, green peppers, Italian sweet red peppers, orange peppers, yellow peppers, eggplants, okra) | cucumber, gourd, asparagus, leek, cherry tomato, garden tomatoes, green bell pepper, Italian sweet red pepper, orange bell pepper, yellow bell pepper, eggplant, okra | cucumber, karela (bitter gourd), torai (sponge gourd), cherry tomatoes, tomatoes, shimla mirch (capsicum; green bell pepper), baigan (brinjal; egg plant), bhindi (okra) | cetriolo, zucca, asparago,  porro, pomodoro iliegino,  pomodoro, peperone (rosso, verde, arancione, giallo), melanzana, gombo (ocra)  (Translation into English: cucumber,  pumpkin, asparagus, leek, cherry tomato,  tomato, pepper (red, green, orange, yellow),  eggplant, okra (ochre)) | キュウリ、カボチャ、スパラガス、ネギ、  トマト、ナス、オクラ、パプリカ、ピーマン (Translation into English: cucumber, pumpkin, asparagus, green onion,  tomato, eggplant, okra,  Paprika, green pepper) |
| Beans and peas | 绿豆、黄腊豆、普通菜豆、蚕豆、绿豆、黑眼豆（豇豆）、长豇豆、红豆、青豆、普通豌豆、鹰嘴豆、角豆、大豆 (Translation into English: Mung beans, yellow wax beans, common kidney beans, broad beans, mung beans, black-eyed peas (cowpeas), long cowpeas, red beans, lima beans, common peas, chickpeas, carob, soybeans) | green beans, yellow wax bean, common bean, broad bean, Mung bean, black-eyed pea (cowpea), yard-long bean, scarlet bean, lima bean, common pea, chickpeas, carob beans, soybean | green beans, pea, broad bean, mung, lobia (black-eye pea; cowpea), rajma (kidney beans), kabuli chana (white chana, chickpeas), soyabean | fagiolini, fagiolini gialli,  fagiolo, fava, fagiolo indiano verde, fagiolo dall'occhio, fagiolo asparago, fagiolo di Spagna, fagiolo di Lima,  piselli, ceci, carruba,  soia  (Translation into English: green beans,  wax beans, bean, broad bean, mung bean, cowpea, yard-long bean,  runner bean, lima bean,  peas, chickpeas, carob,  soybeans) | 大豆、小豆、緑豆、インゲン豆、エンドウ、ソラマメ、ヒヨコ豆、黒目豆（ブラックアイドピー）、黄色サヤインゲンとジュウロクササゲ豆、花豆、イナゴ豆（カロッブ豆）(Translation into English: Soybeans, adzuki beans, mung beans, kidney beans, peas, fava beans, chickpeas, black-eyed peas, yellow string beans and black-eyed peas, flower beans, carob beans) |
| Nuts and seeds | 杏仁、山核桃仁、科罗拉多松子（松子）、巴西坚果、夏威夷果、开心果、榛子、核桃 (Translation into English: Almonds, pecan nuts, Colorado pine nuts (pine nuts), Brazil nuts, macadamia nuts, pistachios, hazelnuts, walnuts) | almond, pecan nut, Colorado pinyon (pine nuts), Brazil nuts, Macadamia nuts, pistachio, hazel nuts, walnut | almond, chilgoza (Colorado pinyon, pine nuts), pista (pistachio), walnut, peanuts | mandorle, noci di Pecan,  pinoli, noci brasiliane,  Macadamia, pistacchi,  nocciole, noci  (Translation into English: almonds,  Pecans, pine nuts, Brazil nuts, macadamia, pistachios, hazelnuts,  walnuts | アーモンド、カボチャの種、栗、ぎんなん、マカデミアナッツ、ピスタチオ、ヘーゼルナッツ、クルミ、ピーカンナッツ、コロラドピニョン（松の実）、ブラジルナッツ (Translation into English: almonds, pumpkin seeds, chestnuts, gingko nuts, macadamia nuts, pistachios, hazelnuts, walnuts, pecans, Colorado piñon (pine nuts), Brazil nuts) |
| Spices and herbs | 迷迭香、百里香、芫荽、甜罗勒、莳萝、藏红花、四季葱、芥菜、甜马郁兰、家独行菜、甜月桂、姜黄、紫花苜蓿、薄荷、芝麻、八角、香菜、陈皮、桂皮、丁香、孜然、生姜、小茴香 (Translation into English: rosemary, thyme, coriander, sweet basil, dill, saffron, green onions, mustard greens, sweet marjoram, house leek, sweet bay, turmeric, alfalfa, mint, sesame seeds, star anise, coriander, tangerine peel, cinnamon, cloves, cumin, ginger, fennel) | rosemary, thyme, coriander, ginger, sweet basil, dill, parsley, sage, rhubarb, saffron, chives, Chinese mustard, sweet marjoram, garden cress, sweet bay, turmeric, alfalfa, peppermint (including their extracted essential oils used as food additives and flavoring) | turmeric, ginger, coriander, jeera (cumin), laung (clove), elaichi (cardamom), rosemary, thyme, saffron, Indian mustard seeds, bay leaves, mint leaves | rosmarino, timo, coriandolo, basilico,  aneto, prezzemolo, salvia,  rabarbaro, zafferano, erba cipollina, senape cinese, maggiorana,  crescione, alloro, curcuma, zenzero, erba medica, menta (Translation into English: rosemary,  thyme, coriander, basil,  dill, parsley, sage, rhubarb, saffron, chives,  Chinese mustard, marjoram, watercress, bay leaves, turmeric, ginger, alfalfa, mint) | 胡椒、わさび、ショウガ、しそ、山椒、胡麻、ミョウガ、唐辛子、八角、ウイキョウ、陳皮、マスタード、ミント、バジル、パクチー（コリアンダー）、パセリ、ディル、チャイブ、ガーデンクレス、スイートマジョラム、サフラン、ローズマリー、セージ、タイム、クミン、シナモン（桂皮）、クローブ（丁子）、ターメリック（ウコン）、月桂樹の葉、ペパーミント (Translation into English: pepper, wasabi, ginger, perilla, Japanese pepper, sesame, ginger, chili pepper, star anise, fennel, chinpi (dried orange peel), mustard, mint, basil, coriander, parsley, dill, chives, garden cress, sweet marjoram, saffron, rosemary, sage, thyme, cumin, cinnamon, cloves, turmeric, bay leaf, peppermint) |
| Cereals and grains | 荞麦、大麦等谷类 (Translation into English: buckwheat, barley and other cereals) | buckwheat, barley | kuttu ka atta (buckwheat), jau (barley) | grano saraceno, orzo, o alimenti che li contengono (Translation into English: buckwheat, barley, or foods containing them) | 大麦（麦飯など）または蕎麦 (Translation into English: barley (such as barley rice) or soba) |
| Other foods | 蘑菇等菌类、橄榄 (Translation into English: mushrooms and other fungi, olives) | mushrooms | mushroom, olive | Funghi (Translation into English: mushrooms) | マッシュルームなどのキノコ類、オリーブ、ケッパー (Translation into English: mushrooms, olives, capers) |
| Fermented foods | 酸菜、酸奶、酱油、泡菜、醋、芝士 (Translation into English: sauerkraut, yogurt, soy sauce, kimchi, vinegar, cheese) | sauerkraut, pickles, kefir, tempeh, natto, kombucha, miso, kimchi, yogurt | dosa, idli, bhatura, buttermilk, curd (yogurt) | crauti, cetriolini, yogurt ma anche kefir, tempeh, natto, kombucha, miso,  kimchi, yogurt (Translation into English: sauerkraut, pickles, yogurt but also kefir, tempeh, natto, kombucha, miso,  kimchi, yogurt) | 納豆、味噌、醤油、キムチ、酢、ぬか漬け、鰹節、ヨーグルト、チーズ、生ハムなど (Translation into English: natto, miso, soy sauce, kimchi, vinegar, rice bran pickles, bonito flakes, yogurt, cheese, prosciutto, etc.) |
| Herbal medicine | 清肺排毒汤、化湿败毒颗粒，宣肺败毒汤，金花清感颗粒，连花清瘟胶囊、血必净注射液 (Translation into English: Qingfei Paidu Decoction, Huashibaidu Granules, Xuanfei Baidu Decoction, Jinhua Qinggan Granules, Lianhua Qingwen Capsules, Xuebijing Injection) | Herbal medicine | ashwagandha, or giloy | medicinali erboristici (Translation into English: herbal medicines) | 補中益気湯、十全大補湯、清肺毒湯、麻黄湯、葛根湯、麻黄附子細辛湯、当帰芍薬散、人参養栄湯、辛夷清肺湯、葛根湯加川芎辛夷など (Hochuekkito, Juzentaihoto, Seishindokuto, Maoto, Kakkonto, Maobusushisishinto, Tokishakuyakusan, Ninjinyoeito, Shini Seiryuto, Kakkonto Kagawashashini: these are names of herbal medicines used for COVID-19 |
|  | 维生素、人参、蜂胶、姜黄丸，软骨素，蜂蜜，灵芝，葡萄籽 (Translation into English: vitamins, ginseng, propolis, turmeric pills, chondroitin, honey, Ganoderma lucidum, grape seed) | vitamins, CBD pills, turmeric pills, propolis | vitamins, CBD pills, turmeric pills, propolis | vitamine, pillole di Cannabidiolo (CBD),  di curcuma, propoli  (Translation into English: vitamins, cannabidiol (CBD) pills,  of turmeric pills, propolis) | ビタミン類や栄養補給飲料 (Translation into English: vitamins and nutritional drinks) |

**Supplementary table 1.** Examples of food items used in each language version of the survey (Part 2. Persian, Polish, Russian, Spanish, and Turkish)

|  | Persian | Polish | Russian | Spanish | Turkish |
| --- | --- | --- | --- | --- | --- |
| Tea | چای سیاه، چای سبز، چای سفید، چای قرمز  (Translation into English: black tea, green tea, white tea, red tea) | czarna, zielona, biała, żółta,  oolong, czerwona, herbata  (Translation into English: black, green,  White, yellow, oolong, red tea) | черный, зеленый, белый,  желтый, чай улун, пуэр или другие (Translation into English: black tea, green tea, white tea, yellow tea, oolong tea, puerh tea or others) | té negro, té verde, té blanco, té amarillo, té oolong o azul, té rojo u otros (Translation into English: black tea, green Tea, white tea, yellow tea, oolong or blue tea, red tea or others) | siyah çay, yeşil çay, beyaz çay (Translation into English:  black tea, green tea, white tea) |
| Herbal tea | چای زنجبیلی، چای لیمو، چای بیان  (Translation into English: ginger tea, lemon tea, licorice tea) | Imbir, trawa cytrynowa, lukrecja  (Translation into English: ginger tea,  Lemongrass tea, licorice tea) | имбиря, лимонного сорго (лемонграсс), лакрицы (солодки), гибискуса (каркаде), ромашки,  мяты или других (Translation into English: ginger tea, lemongrass tea, licorice tea, hibiscus tea, chamomile tea, mint tea or others) | de jengibre, de hierba luisa, de regaliz (Translation into English: ginger tea, lemon verbena tea, licorice tea) | Ihlamur, ada çayı  (Translation into English: Linden tea, sage tea) |
| Coffee | قهوه  (Translation into English: coffee) | Kawa  (Translation into English: coffee) | Кофе  (Translation into English: coffee) | Café  (Translation into English: coffee) | Kahve  (Translation into English: coffee) |
| Hot chocolate | کاکائو  (Translation into English: cocoa) | Kakao  (Translation into English: cocoa) | натуральный какао (Translation into English: cocoa) | Cacao  (Translation into English: cocoa) | Kakao  (Translation into English: cocoa) |
| Cider | Not included in Persian version | Cydr  (Translation into English: cider) | Сидр  (Translation into English: cider) | zumo de manzana (Translation into English: apple juice) | elma suyu  (Translation into English: apple juice) |
| Alcohol | Not included in Persian version | wino, piwo, whisky, brandy (Translation into English: wine, beer, whiskey, brandy | вино, пиво, виски, в одка или другие (Translation into English: wine, beer, whiskey, vodka or others) | vino, cerveza, whisky, brandy u otros (Translation into English: wine, beer, whiskey, brandy or others) | bira, şarap, rakı, v.d  (Translation into English: beer, wine,  raki, etc.) |

**Supplementary Table 2**. Comparison of the severity of symptoms reported by “Not tested” and “Tested positive” groups.

| Symptoms |  |  |  |
| --- | --- | --- | --- |
| Fever | χ2=0.088 | df=2 | P=0.957 |
| Headache | χ2=20.244 | df=4 | P=0.0004 |
| Chills | χ2=1.334 | df=4 | P=0.856 |
| Lightheadedness | χ2=19.026 | df=4 | P=0.0007 |
| dizziness | χ2=0.876 | df=4 | P=0.928 |
| body aches | χ2=3.214 | df=4 | P=0.523 |
| Memory loss | χ2=20.993 | df=4 | P=0.0003 |
| fatigue | χ2=5.737 | df=4 | P=0.220 |
| coughs | χ2=7.210 | df=4 | P=0.1251 |
| shortness of breaths | χ2=4.460 | df=4 | P=0.347 |
| runny nose/nose congestion | χ2=3.487 | df=4 | P=0.480 |
| Diarrhea | χ2=12.372 | df=4 | P=0.015 |
| nausea | χ2=0.722 | df=4 | P=0.949 |
| hair loss | χ2=0.463 | df=1 | P=0.496 |
| Smell dysfunction | χ2=2.122 | df=2 | P=0.713 |
| Taste dysfunction | χ2=4.012 | df=2 | P=0.134 |
| Chemesthesis dysfunction | χ2=2.532 | df=2 | P=0.639 |
| other symptoms | χ2=2.679 | df=4 | P=0.613 |

**Supplementary Table 3**. Comparison of the food and beverage consumption reported by “Not tested”, “Tested positive”, and “Did not get COVID” groups.

| Foods and beverage types | N | Chi-square value (df=6) | n (%) | | | | P | Cramer’s V (Effect size)  df=2  *: small association (0.07 < 0.21)  **: moderate association (0.21 < 0.35) |
| --- | --- | --- | --- | --- | --- | --- | --- | --- |
|  |  | χ2 | Daily | Weekly | Monthly | Never |  |  |
| Tea  Tested+  Not tested  Did not get COVID | 1461 | χ2=54.528 | 319 (41.97)  168 (62.22)  243 (56.38) | 119 (15.66)  28 (10.37)  72 (16.71) | 69 (9.08)  8 (2.96)  21 (4.87) | 253 (33.29)  66 (24.44)  95 (22.04) | P<0.001 | 0.14* |
| Herbal tea  Tested+  Not tested  Did not get COVID | 1461 | χ2=31.060 | 136 (18.87)  76 (28.15)  62 (14.42) | 133 (17.48)  41 (15.19)  59 (13.72) | 81 (10.64)  27 (10.00)  70 (16.28) | 411 (54.10)  126 (47.67)  239 (55.58) | P<0.001 | 0.10* |
| Coffee  Tested+  Not tested  Did not get COVID | 1459 | χ2=22.207 | 415 (53.90)  115 (42.75)  230 (54.76) | 98 (12.73)  33 (12.27)  52 (12.38) | 41 (5.32)  17 (6.32)  36 (8.57) | 216 (28.05)  104 (38.66)  102 (24.29) | P=0.0011 | 0.087* |
| Apple cider  Tested+  Not tested  Did not get COVID | 1277 | χ2=79.553 | 15 (2.14)  5 (2.69)  32 (8.21) | 20 (2.85)  4 (2.15)  48 (12.31) | 69 (9.84)  18 (9.68)  46 (11.79) | 597 (85.16)  159 (85.48)  264 (67.69) | P<0.001 | 0.18* |
| Hot chocolate  Tested+  Not tested  Did not get COVID | 1446 | χ2=9.771 | 43 (5.70)  6 (2.26)  18 (4.24) | 72 (9.54)  22 (8.27)  44 (10.35) | 129 (17.09)  41 (15.41)  57 (13.41) | 511 (67.68)  197 (74.06)  306 (72.00) | P=0.135 | 0.057 |
| Alcohol  Tested+  Not tested  Did not get COVID | 1277 | χ2=23.920 | 57 (8.13)  21 (11.29)  69 (17.69) | 165 (23.54)  44 (23.66)  75 (19.23) | 153 (21.83)  35 (18.82)  73 (18.72) | 326 (46.50)  86 (46.24)  173 (44.36) | P<0.001 | 0.097* |
| Citrus fruits  Tested+  Not tested  Did not get COVID | 1424 | χ2=31.541 | 211 (28.36)  108 (41.38)  93 (22.20) | 290 (38.98)  85 (32.57)  164 (39.14) | 119 (15.99)  29 (11.11)  77 (18.38) | 124 (16.67)  39 (14.94)  85 (20.29) | P<0.001 | 0.105* |
| Berries  Tested+  Not tested  Did not get COVID | 1418 | χ2=21.167 | 65 (8.75)  17 (6.56)  30 (7.21) | 229 (30.82)  60 (23.17)  120 (28.85) | 191 (25.71)  52 (20.08)  113 (27.16) | 258 (34.72)  130 (50.19)  153 (36.78) | P=0.0017 | 0.086* |
| Pome fruits  Tested+  Not tested  Did not get COVID | 1412 | χ2=25.777 | 183 (24.66)  78 (30.35)  98 (23.73) | 298 (40.16)  103 (40.08)  128 (30.99) | 130 (17.52)  27 (10.51)  86 (20.82) | 131 (17.65)  49 (19.07)  101 (24.46) | P<0.001 | 0.096* |
| Stone fruits  Tested+  Not tested  Did not get COVID | 1403 | χ2=8.617 | 60 (8.13)  28 (11.07)  35 (8.50) | 204 (27.64)  66 (26.09)  96 (23.30) | 213 (28.86)  57 (22.53)  119 (28.88) | 261 (35.37)  102 (40.32)  162 (39.32) | N.S. | 0.055 |
| Tropical fruits  Tested+  Not tested  Did not get COVID | 1397 | χ2=8.001 | 176 (23.95)  59 (23.32)  90 (22.00) | 316 (42.99)  107 (42.29)  153 (37.41) | 137 (18.64)  43 (17.00)  91 (22.25) | 106 (14.42)  44 (17.39)  75 (18.34) | P=0.24 | 0.054 |
| Other fruits  Tested+  Not tested  Did not get COVID | 1390 | χ2=16.516 | 63 (8.61)  34 (13.39)  39 (9.65) | 255 (34.84)  93 (36.61)  122 (30.20) | 233 (31.83)  57 (22.44)  117 (28.96) | 181 (24.73)  70 (27.56)  126 (31.19) | P<0.05 | 0.077* |
| Leaf vegetables  Tested+  Not tested  Did not get COVID | 1379 | χ2=23.957 | 287 (39.42)  92 (36.80)  207 (51.62) | 285 (39.15)  99 (39.60)  112 (27.93) | 79 (10.85)  34 (13.60)  48 (11.97) | 77 (10.58)  25 (10.00)  34 (8.48) | P<0.001 | 0.093* |
| Root vegetables  Tested+  Not tested  Did not get COVID | 1371 | χ2=15.828 | 270 (37.45)  111 (44.22)  194 (48.62) | 353 (48.96)  103 (41.04)  153 (38.35) | 54 (7.49)  19 (7.57)  28 (7.02) | 44 (6.10)  18 (7.17)  24 (6.02) | P<0.05 | 0.076* |
| Other vegetables  Tested+  Not tested  Did not get COVID | 1358 | χ2=13.848 | 294 (41.06)  106 (42.57)  189 (48.09) | 330 (46.09)  103 (41.37)  157 (39.95) | 53 (7.40)  17 (6.83)  32 (8.14) | 39 (5.45)  23 (9.24)  15 (3.82) | P<0.05 | 0.071* |
| Beans and peas  Tested+  Not tested  Did not get COVID | 1354 | χ2=47.242 | 88 (12.34)  45 (18.00)  108 (27.62) | 396 (55.54)  132 (52.80)  164 (41.94) | 113 (15.85)  43 (17.20)  71 (18.16) | 116 (16.27)  30 (12.00)  48 (12.28) | P<0.001 | 0.132* |
| Nuts and seeds  Tested+  Not tested  Did not get COVID | 1351 | χ2=10.160 | 168 (23.66)  66 (26.51)  90 (22.96) | 284 (40.00)  83 (33.33)  130 (33.16) | 148 (20.85)  55 (22.09)  89 (22.70) | 110 (15.49)  45 (18.07)  83 (21.17) | N.S. | 0.061 |
| Spices and herbs  Tested+  Not tested  Did not get COVID | 1340 | χ2=35.757 | 271 (38.28)  119 (48.77)  208 (53.61) | 254 (35.88)  63 (25.82)  88 (22.68) | 57 (8.05)  24 (9.84)  42 (10.82) | 126 (17.80)  38 (15.58)  50 (12.89) | P<0.001 | 0.117* |
| Cereals and grains including barley  Tested+  Not tested  Did not get COVID | 1318 | χ2=21.060 | 190 (27.34)  54 (22.31)  91 (23.88) | 206 (29.64)  69 (28.51)  119 (31.23) | 92 (13.24)  33 (13.64)  81 (21.26) | 207 (29.78)  86 (35.54)  90 (23.62) | P=0.0018 | 0.089* |
| Other foods such as mushrooms  Tested+  Not tested  Did not get COVID | 1327 | χ2=51.960 | 41 (5.87)  9 (3.72)  62 (16.02) | 239 (34.24)  63 (26.03)  129 (33.33) | 236 (33.81)  97 (40.08)  109 (28.17) | 182 (26.07)  73 (30.17)  87 (22.48) | P<0.001 | 0.140* |
| Fermented foods  Tested+  Not tested  Did not get COVID | 1331 | χ2=62.945 | 169 (24.07)  64 (26.23)  178 (46.23) | 223 (31.77)  68 (27.87)  83 (21.56) | 80 (11.40)  27 (11.07)  39 (10.13) | 230 (32.76)  85 (34.84)  85 (22.08) | P<0.001 | 0.154* |
| Herbal medicines  Tested+  Not tested  Did not get COVID | 1331 | χ2=18.872 | 107 (15.24)  45 (18.44)  31 (8.05) | 63 (8.97)  28 (11.48)  35 (9.09) | 43 (6.13)  15 (6.15)  27 (7.01) | 489 (69.66)  156 (63.93)  292 (75.84) | P=0.0044 | 0.0842* |
| Supplements  Tested+  Not tested  Did not get COVID | 1314 | χ2=38.246 | 235 (33.96)  83 (34.16)  78 (20.58) | 64 (9.25)  28 (11.53)  29 (7.65) | 41 (5.92)  18 (7.41)  15 (3.96) | 352 (50.87)  114 (46.91)  257 (67.81) | P<0.001 | 0.121* |
| Essential oils for prevention  Tested+  Not tested  Did not get COVID | 1315 | χ2=10.904 | 76 (11.01)  22 (9.09)  36 (9.40) | 48 (6.96)  16 (6.61)  20 (5.22) | 24 (3.48)  2 (0.83)  20 (5.22) | 542 (78.55)  202 (83.47)  307 (80.16) | N.S. | 0.064 |
| Essential oils for recovery  Tested+  Not tested | 926 | χ2=4.807  df=3 | 112 (16.33)  29 (12.08) | 51 (7.43)  17 (7.08) | 23 (3.35)  4 (1.67) | 500 (72.89)  190 (79.17) | N.S. | 0.072*  df=1 |

**Supplementary Table 4.** Ethnicity of the participants who answered each language version of the survey

| Language of the survey | Ethnicity of the participants |
| --- | --- |
| Chinese | Not clear (none wrote answers) |
| English | Greek, Asian, Indian, Vietnam, Israeli, Irish/German, Bosnian, Montenegrin, not clear |
| Indian English | 100% Indian |
| Italian | 97.3% Italian, 2.7% not clear |
| Japanese | 98.3% Japanese, 1.1% Chinese, 0.6% American |
| Persian | 100% Iranian |
| Polish | Not clear (15 wrote answers, of which 9 were in Polish, others unclear) |
| Russian | 91.3% Russian, 8.7% not clear |
| Spanish | 94.2% Spanish, 5.8% not clear |
| Turkish | Not clear (only 2 wrote answers) |

**3 Supplementary Material**

**English version of the survey**

GCCR - Phytochemicals - English1

Q82 I am at least 18 years old, and I wish to voluntarily participate in this study.

o Yes (1)

o No (2)

Skip To: End of Survey If I am at least 18 years old, and I wish to voluntarily participate in this study. = No

End of Block: Block 1

Start of Block: Block 2

During the pandemic, many people have tried various methods to help accelerate their recovery from symptoms of COVID-19. Among the most popular approaches are at home remedies and herbal medicines. It is still unclear whether some foods, drinks, essential oils, and their ingredients may be helpful in recovering from COVID-19.
 In this survey, we would like to hear if you tried any specific foods, drinks, home remedies, or herbal medicines that you have heard or think work in suppressing COVID-19 symptoms and/or in facilitating recovery. If you did, please let us know – we would like to hear their effects.
 Thank you so much for your voluntary participation. The data will be de-identified, saved and protected in our system.

 – Global Consortium for Chemosensory Research Team


 Please let us know if you have any questions regarding the study.
 General contact: Dr. Sachiko Koyama, Indiana University, +1-812-345-6155 sakoyama@iu.edu


 One of the lead researchers has a company that does research and development of products using phytochemicals. This company is not funding or sponsoring this research study. We are giving you this information so you can decide if this affects your willingness to participate in this study.

Q2 Did you get COVID-19?

o Not tested, but I suspect that I got it (1)

o Yes – tested positive via PCR or antibody test (2)

o No or not aware (3)

Display This Question:

If Did you get COVID-19? = Not tested, but I suspect that I got it

Or Did you get COVID-19? = Yes – tested positive via PCR or antibody test

Q3 If yes, what symptoms did/do you have (overall and when at worst)?

|  | No Fever (1) | Mild Fever (2) | High Fever (3) |
| --- | --- | --- | --- |
| Fever (measured or estimated): (1) | o | o | o |

Display This Question:

If Did you get COVID-19? = Not tested, but I suspect that I got it

Or Did you get COVID-19? = Yes – tested positive via PCR or antibody test

Q4 (Continued)

|  | No (1) | Mild (2) | Moderate (3) | Severe (4) | Very Severe (5) |
| --- | --- | --- | --- | --- | --- |
| Headache (1) | o | o | o | o | o |
| Cough (2) | o | o | o | o | o |
| Shortness of breath (3) | o | o | o | o | o |
| Runny nose or congestion (4) | o | o | o | o | o |
| Muscle/body aches/soreness (5) | o | o | o | o | o |
| Chills (6) | o | o | o | o | o |
| Light headedness or brain fog (7) | o | o | o | o | o |
| Dizziness (8) | o | o | o | o | o |
| Nausea (9) | o | o | o | o | o |
| Fatigue (10) | o | o | o | o | o |
| Memory loss, confusion, hallucination (11) | o | o | o | o | o |
| Diarrhea, gastrointestinal symptoms (12) | o | o | o | o | o |
| Other symptoms (13) | o | o | o | o | o |

Display This Question:

If Did you get COVID-19? = Not tested, but I suspect that I got it

Or Did you get COVID-19? = Yes – tested positive via PCR or antibody test

Q5 (Continued)

|  | Yes (1) | No (2) |
| --- | --- | --- |
| Hair loss (1) | o | o |

Display This Question:

If Did you get COVID-19? = Not tested, but I suspect that I got it

Or Did you get COVID-19? = Yes – tested positive via PCR or antibody test

Q6 (Continued)

|  | No loss (1) | Reduced sensation (2) | Total loss of sensation (3) |
| --- | --- | --- | --- |
| Loss of smell or reduced smell (1) | o | o | o |
| Loss of taste or reduced taste (2) | o | o | o |
| Loss of chemesthesis or reduced chemesthesis (includes the burning, stinging, pricking sense associated with the spiciness of chili or the freshness/coolness of menthol): (3) | o | o | o |

Display This Question:

If (Continued) = Loss of taste or reduced taste [ Reduced sensation ]

Or (Continued) = Loss of taste or reduced taste [ Total loss of sensation ]

Q7 If you experienced reduced ability to taste or total loss of taste, which sensation?

▢ Sweet (1)

▢ Sour (2)

▢ Salty (3)

▢ Bitter (4)

▢ Umami (savory) (5)

Display This Question:

If Did you get COVID-19? = Yes – tested positive via PCR or antibody test

Or Did you get COVID-19? = Not tested, but I suspect that I got it

Q8 When did you have COVID for the first time (in case you got it multiple times)?

o Before February 2020 (1)

o Between March to May, 2020 (2)

o Between June to August, 2020 (3)

o Between September to November, 2020 (4)

o Between December 2020 to February 2021 (5)

o Between March to May, 2021 (6)

o June to August, 2021 (7)

o September to November, 2021 (8)

o After December 2021 (9)

Display This Question:

If Did you get COVID-19? = Yes – tested positive via PCR or antibody test

Or Did you get COVID-19? = Not tested, but I suspect that I got it

Q9 Approximately how many days did it take to have a negative COVID test, or how many days did it take to recover from the worst phase (if you did not get tested)?

________________________________________________________________

Display This Question:

If Did you get COVID-19? = Yes – tested positive via PCR or antibody test

Or Did you get COVID-19? = Not tested, but I suspect that I got it

Q10 Do (did) you have persisting symptoms of COVID-19?

o Yes (1)

o No (2)

Display This Question:

If Do (did) you have persisting symptoms of COVID-19? = Yes

Q12 Which persisting symptoms do you have? Choose all that apply

▢ Fever (1)

▢ Headache (2)

▢ Cough (3)

▢ Shortness of breath (4)

▢ Runny nose or congestion (5)

▢ Muscle/body aches/soreness (6)

▢ Chills (7)

▢ Light headedness or brain fog (8)

▢ Dizziness (9)

▢ Nausea (10)

▢ Fatigue (11)

▢ Hair loss (12)

▢ Memory loss (13)

▢ Confusion (14)

▢ Hallucination (15)

▢ Loss or reduced smell (16)

▢ Loss or reduced taste (17)

▢ Loss or reduced chemesthesis (18)

▢ Diarrhea (19)

▢ Gastrointestinal Symptoms (20)

▢ Others (21)

Q13 Please answer to the following questions about your fluids/beverage consumption during the pandemic (since December 2019).

Q14 During the pandemic, how often did you drink tea such as black tea, green tea, white tea, yellow tea, oolong tea, red tea, others? (Note: This excludes Kombucha. Please check one answer that best describes the frequency of your use of the food category)

o Never (1)

o Daily (2)

o Weekly (3)

o Monthly (4)

Q15 During the pandemic, how often did you drink herbal tea such as ginger, lemongrass, licorice, others?

o Never (1)

o Daily (2)

o Weekly (3)

o Monthly (4)

Display This Question:

If During the pandemic, how often did you drink herbal tea such as ginger, lemongrass, licorice, oth... = Daily

Or During the pandemic, how often did you drink herbal tea such as ginger, lemongrass, licorice, oth... = Weekly

Or During the pandemic, how often did you drink herbal tea such as ginger, lemongrass, licorice, oth... = Monthly

Q16 What type of herbal tea do/did you drink?

▢ Ginger (11)

▢ Lemongrass (14)

▢ Others (15) __________________________________________________

Q17 During the pandemic, how often did you drink coffee?

o Never (1)

o Daily (2)

o Weekly (3)

o Monthly (4)

Display This Question:

If During the pandemic, how often did you drink coffee? = Daily

Or During the pandemic, how often did you drink coffee? = Weekly

Or During the pandemic, how often did you drink coffee? = Monthly

Q18 What type of coffee do/did you drink?

________________________________________________________________

Q19 During the pandemic, how often did you drink cocoa?

o Never (1)

o Daily (2)

o Weekly (3)

o Monthly (4)

Q20 During the pandemic, how often did you drink cider?

o Never (1)

o Daily (2)

o Weekly (3)

o Monthly (4)

Q21 During the pandemic, how often did you drink alcohol such as wine, beer, whiskey, brandy, others?

o Never (1)

o Daily (2)

o Weekly (3)

o Monthly (4)

Display This Question:

If During the pandemic, how often did you drink alcohol such as wine, beer, whiskey, brandy, others? = Daily

Or During the pandemic, how often did you drink alcohol such as wine, beer, whiskey, brandy, others? = Weekly

Or During the pandemic, how often did you drink alcohol such as wine, beer, whiskey, brandy, others? = Monthly

Q22 What type of alcoholic beverage do/did you drink? You may write more than one.

▢ beer (4)

▢ brandy (5)

▢ whiskey (6)

▢ wine (7)

▢ Others (8) __________________________________________________

Q77 Did you change your fluid/beverage habits after the pandemic started?

o Yes (1)

o No (2)

Display This Question:

If Did you change your fluid/beverage habits after the pandemic started? = Yes

Q80 Why did you change your fluid/beverage habits?

▢ To prevent contracting the virus, I started to drink medicinal/herbal tea more often. (1)

▢ Regular liquids did not taste good anymore. (2)

▢ No specific reason (3)

▢ Other reason (4)

Display This Question:

If Did you change your fluid/beverage habits after the pandemic started? = Yes

Q78 Name of the drink

________________________________________________________________

Q23 Please tell us if there are specific foods that you used during this pandemic. Fruits (check one answer that best describes the frequency of your use of the food category)

Q24 Citrus fruits such as mandarin orange, clementine, tangerine, lemon, lime, pummelo, kumquat, sweet orange?

o Never (1)

o Daily (2)

o Weekly (3)

o Monthly (4)

Display This Question:

If Citrus fruits such as mandarin orange, clementine, tangerine, lemon, lime, pummelo, kumquat, swee... = Daily

Or Citrus fruits such as mandarin orange, clementine, tangerine, lemon, lime, pummelo, kumquat, swee... = Weekly

Or Citrus fruits such as mandarin orange, clementine, tangerine, lemon, lime, pummelo, kumquat, swee... = Monthly

Q25 What citrus fruit do/did you eat?

▢ clementine (4)

▢ kumquat (5)

▢ lemon (6)

▢ lime (7)

▢ mandarin orange (8)

▢ pummelo (9)

▢ sweet orange (10)

▢ tangerine (11)

▢ others (12) __________________________________________________

Q26 Berries such as cranberry, blueberry, huckleberry, strawberry, blackberry, raspberry, black elderberry, gooseberry, black mulberry, pitanga, black currants (cassis)?

o Never (1)

o Daily (2)

o Weekly (3)

o Monthly (4)

Display This Question:

If Berries such as cranberry, blueberry, huckleberry, strawberry, blackberry, raspberry, black elder... = Daily

Or Berries such as cranberry, blueberry, huckleberry, strawberry, blackberry, raspberry, black elder... = Weekly

Or Berries such as cranberry, blueberry, huckleberry, strawberry, blackberry, raspberry, black elder... = Monthly

Q27 What berries do you eat?

▢ blackberry (4)

▢ black currants (cassis) (5)

▢ black elderberry (6)

▢ black mulberry (7)

▢ blueberry (8)

▢ cranberry (9)

▢ gooseberry (10)

▢ huckleberry (11)

▢ pitanga (12)

▢ raspberry (13)

▢ strawberry (14)

▢ others (15) __________________________________________________

Q84 Pome fruits such as apple, pear, quince?

o Never (1)

o Daily (2)

o Weekly (3)

o Monthly (4)

Display This Question:

If Pome fruits such as apple, pear, quince? = Daily

Or Pome fruits such as apple, pear, quince? = Weekly

Or Pome fruits such as apple, pear, quince? = Monthly

Q85 What pome fruits did you eat?

▢ apple (4)

▢ pear (5)

▢ quince (6)

▢ others (7) __________________________________________________

Q86 Stone fruits such as plum, cherry, apricot, peach?

o Never (1)

o Daily (2)

o Weekly (3)

o Monthly (4)

Display This Question:

If Stone fruits such as plum, cherry, apricot, peach? = Daily

Or Stone fruits such as plum, cherry, apricot, peach? = Weekly

Or Stone fruits such as plum, cherry, apricot, peach? = Monthly

Q87 What stone fruits did you eat?

▢ apricot (4)

▢ cherry (5)

▢ peach (6)

▢ plum (7)

▢ others (8) __________________________________________________

Q28 Tropical fruits such as banana, mango, papaya, kiwi, pineapple, date, avocado, soursop?

o Never (1)

o Daily (2)

o Weekly (3)

o Monthly (4)

Display This Question:

If Tropical fruits such as banana, mango, papaya, kiwi, pineapple, date, avocado, soursop? = Daily

Or Tropical fruits such as banana, mango, papaya, kiwi, pineapple, date, avocado, soursop? = Weekly

Or Tropical fruits such as banana, mango, papaya, kiwi, pineapple, date, avocado, soursop? = Monthly

Q29 What tropical fruit do/did you eat? ( )

▢ avocado (4)

▢ banana (5)

▢ date (6)

▢ kiwi (7)

▢ mango (8)

▢ papaya (9)

▢ pineapple (10)

▢ soursop (11)

▢ others (12) __________________________________________________

Q30 Other fruits such as fig, muskmelon, watermelon, and grapes?

o Never (1)

o Daily (2)

o Weekly (3)

o Monthly (4)

Display This Question:

If Other fruits such as fig, muskmelon, watermelon, and grapes? = Daily

Or Other fruits such as fig, muskmelon, watermelon, and grapes? = Weekly

Or Other fruits such as fig, muskmelon, watermelon, and grapes? = Monthly

Q31 What other fruits do/did you eat?

▢ fig (4)

▢ grapes (5)

▢ muskmelon (6)

▢ watermelon (7)

▢ others (8) __________________________________________________

Q32 Leaf vegetables such as lettuce, chicory, endive, turnip leaves, spinach, celery, purslane, cabbage, broccoli, brussel sprouts, cauliflower, beet leaves, fennel, arugula, green parts (leaves) of onions, and artichokes?

o Never (1)

o Daily (2)

o Weekly (3)

o Monthly (4)

Display This Question:

If Leaf vegetables such as lettuce, chicory, endive, turnip leaves, spinach, celery, purslane, cabba... = Daily

Or Leaf vegetables such as lettuce, chicory, endive, turnip leaves, spinach, celery, purslane, cabba... = Weekly

Or Leaf vegetables such as lettuce, chicory, endive, turnip leaves, spinach, celery, purslane, cabba... = Monthly

Q33 What leaf vegetables do/did you eat?

▢ arugula (4)

▢ beet leaves (5)

▢ broccoli (6)

▢ brussel sprouts (7)

▢ cabbage (8)

▢ cauliflower (9)

▢ celery (10)

▢ chicory (11)

▢ endive (12)

▢ fennel (13)

▢ green onions (14)

▢ lettuce (15)

▢ purslane (16)

▢ spinach (17)

▢ turnip leaves (18)

▢ artichokes (19)

▢ others (20) __________________________________________________

Q34 Root vegetables such as carrot, turnip, garlic, beetroots, parsnip roots, parsley root, radish, onion, sacred lotus, taro, yam, sweet potato, potato?

o Never (1)

o Daily (2)

o Weekly (3)

o Monthly (4)

Display This Question:

If Root vegetables such as carrot, turnip, garlic, beetroots, parsnip roots, parsley root, radish, o... = Daily

Or Root vegetables such as carrot, turnip, garlic, beetroots, parsnip roots, parsley root, radish, o... = Weekly

Or Root vegetables such as carrot, turnip, garlic, beetroots, parsnip roots, parsley root, radish, o... = Monthly

Q35 What root vegetables do/did you eat?

▢ beetroots (5)

▢ carrot (6)

▢ garlic (7)

▢ onion (9)

▢ parsley roots (10)

▢ parsnip roots (11)

▢ potato (12)

▢ radish (13)

▢ sacred lotus (14)

▢ sweet potato (15)

▢ taro (16)

▢ turnip (18)

▢ yam (19)

▢ others (20) __________________________________________________

Q88 Other vegetables such as cucumber, gourd, asparagus, leek, cherry tomato, garden tomatoes, green bell pepper, Italian sweet red pepper, orange bell pepper, yellow bell pepper, eggplant, okra?

o Never (1)

o Daily (2)

o Weekly (3)

o Monthly (4)

Display This Question:

If Other vegetables such as cucumber, gourd, asparagus, leek, cherry tomato, garden tomatoes, green... = Daily

Or Other vegetables such as cucumber, gourd, asparagus, leek, cherry tomato, garden tomatoes, green... = Weekly

Or Other vegetables such as cucumber, gourd, asparagus, leek, cherry tomato, garden tomatoes, green... = Monthly

Q89 What other vegetables do/did you eat?

▢ asparagus (4)

▢ cherry tomatoes (5)

▢ cucumber (6)

▢ gourd (7)

▢ eggplant (8)

▢ garden tomatoes (9)

▢ green bell pepper (10)

▢ Italian sweet red pepper (11)

▢ leek (12)

▢ okra (13)

▢ orange bell pepper (14)

▢ yellow bell pepper (15)

▢ others (16) __________________________________________________

Q36 Beans and peas such as green beans, yellow wax bean, common bean, broad bean, Mung bean, black-eyed pea (cowpea), yardlong bean, scarlet bean, lima bean, common pea, chickpeas, carob beans, soybean?

o Never (1)

o Daily (2)

o Weekly (3)

o Monthly (4)

Display This Question:

If Beans and peas such as green beans, yellow wax bean, common bean, broad bean, Mung bean, black-ey... = Daily

Or Beans and peas such as green beans, yellow wax bean, common bean, broad bean, Mung bean, black-ey... = Weekly

Or Beans and peas such as green beans, yellow wax bean, common bean, broad bean, Mung bean, black-ey... = Monthly

Q37 What beans and peas do/did you eat?

▢ black-eyed pea (cowpea) (4)

▢ broad bean (5)

▢ carob beans (6)

▢ chickpeas (7)

▢ common beans (8)

▢ common peas (9)

▢ green beans (10)

▢ lima bean (11)

▢ Mung bean (12)

▢ scarlet beans (13)

▢ soybeans (14)

▢ yellow wax beans (15)

▢ yardlong bean (16)

▢ others (17) __________________________________________________

Q38 Nuts and seeds such as almond, pecan nut, Colorado pinyon (pine nuts), Brazil nuts, Macadamia nuts, pistachio, hazel nuts, walnut?

o Never (1)

o Daily (2)

o Weekly (3)

o Monthly (4)

Display This Question:

If Nuts and seeds such as almond, pecan nut, Colorado pinyon (pine nuts), Brazil nuts, Macadamia nut... = Daily

Or Nuts and seeds such as almond, pecan nut, Colorado pinyon (pine nuts), Brazil nuts, Macadamia nut... = Weekly

Or Nuts and seeds such as almond, pecan nut, Colorado pinyon (pine nuts), Brazil nuts, Macadamia nut... = Monthly

Q39 What nuts and seeds do/did you eat

▢ almond (4)

▢ Brazil nuts (5)

▢ Colorado pinyon (pine nuts) (6)

▢ hazel nuts (7)

▢ Macadamia nuts (8)

▢ pecan nut (9)

▢ pistachio (10)

▢ walnuts (11)

▢ others (12) __________________________________________________

Q40 Spices and herbs such as rosemary, thyme, coriander, ginger, sweet basil, dill, parsley, sage, rhubarb, saffron, chives, Chinese mustard, sweet marjoram, garden cress, sweet bay, turmeric, alfalfa, peppermint (including their extracted essential oils used as food additives and flavoring)?

o Never (1)

o Daily (2)

o Weekly (3)

o Monthly (4)

Display This Question:

If Spices and herbs such as rosemary, thyme, coriander, ginger, sweet basil, dill, parsley, sage, rh... = Daily

Or Spices and herbs such as rosemary, thyme, coriander, ginger, sweet basil, dill, parsley, sage, rh... = Weekly

Or Spices and herbs such as rosemary, thyme, coriander, ginger, sweet basil, dill, parsley, sage, rh... = Monthly

Q41 What spices and herbs do/did you eat?

▢ alfalfa (4)

▢ Chinese mustard (5)

▢ chives (6)

▢ coriander (7)

▢ dill (8)

▢ garden cress (9)

▢ ginger (29)

▢ parsley (10)

▢ peppermint (11)

▢ rhubarb (12)

▢ rosemary (13)

▢ saffron (14)

▢ sage (15)

▢ sweet basil (16)

▢ sweet bay (17)

▢ sweet marjoram (18)

▢ thyme (19)

▢ turmeric (20)

▢ others (21) __________________________________________________

Q42 Cereals/grains such as or using buckwheat, barley?

o Never (1)

o Daily (2)

o Weekly (3)

o Monthly (4)

Display This Question:

If Cereals/grains such as or using buckwheat, barley? = Daily

Or Cereals/grains such as or using buckwheat, barley? = Weekly

Or Cereals/grains such as or using buckwheat, barley? = Monthly

Q43 What buckwheat and barley food do/did you eat? ( )

________________________________________________________________

Q44 Other foods such as mushroom?

o Never (1)

o Daily (2)

o Weekly (3)

o Monthly (4)

Display This Question:

If Other foods such as mushroom? = Daily

Or Other foods such as mushroom? = Weekly

Or Other foods such as mushroom? = Monthly

Q45 What other foods do/did you eat?

▢ mushroom (4)

▢ Others (6) __________________________________________________

Q46 Please tell us how frequently you ate/drank fermented foods during this pandemic to prevent catching COVID-19 or to facilitate recovery from COVID-19, such as sauerkraut, pickles, kefir, tempeh, natto, kombucha, miso, kimchi, yogurt? (check one answer that best describes the frequency of your use of the food category)

o Never (1)

o Daily (2)

o Weekly (3)

o Monthly (4)

Display This Question:

If Please tell us how frequently you ate/drank fermented foods during this pandemic to prevent catch... = Daily

Or Please tell us how frequently you ate/drank fermented foods during this pandemic to prevent catch... = Weekly

Or Please tell us how frequently you ate/drank fermented foods during this pandemic to prevent catch... = Monthly

Q47 What fermented foods/drinks do you eat/drink?

▢ kefir (4)

▢ kimchi (5)

▢ kombucha (6)

▢ miso (7)

▢ natto (8)

▢ pickles (9)

▢ sauerkraut (10)

▢ tempeh (11)

▢ yogurt (12)

▢ others (13) __________________________________________________

Display This Question:

If Please tell us how frequently you ate/drank fermented foods during this pandemic to prevent catch... = Daily

And Please tell us how frequently you ate/drank fermented foods during this pandemic to prevent catch... = Weekly

And Please tell us how frequently you ate/drank fermented foods during this pandemic to prevent catch... = Monthly

Q48 What food materials/ingredients are they made from?

________________________________________________________________

Q49 Please tell us how frequently you took **herbal medicines** during this pandemic **to prevent catching COVID-19 or facilitate recovery from COVID-19.** (check one answer that best describes the frequency of your use of the food category)

o Never (1)

o Daily (2)

o Weekly (3)

o Monthly (4)

Display This Question:

If Please tell us how frequently you took herbal medicines during this pandemic to prevent catching... = Daily

Or Please tell us how frequently you took herbal medicines during this pandemic to prevent catching... = kly

Or Please tell us how frequently you took herbal medicines during this pandemic to prevent catching... = Monthly

Q50 Which herbal medicine(s) did you take?

________________________________________________________________

Q51 Please tell us how frequently you took any **supplements** like vitamins, CBD pills, turmeric pills, propolis that you used during this pandemic **to prevent catching COVID-19 or facilitate recovery from COVID-19.** (check one answer that best describes the frequency of your use of the food category)

o Never (1)

o Daily (2)

o Weekly (3)

o Monthly (4)

Display This Question:

If Please tell us how frequently you took any supplements like vitamins, CBD pills, turmeric pills,... = Daily

Or Please tell us how frequently you took any supplements like vitamins, CBD pills, turmeric pills,... = Weekly

Or Please tell us how frequently you took any supplements like vitamins, CBD pills, turmeric pills,... = Monthly

Q52 Which supplement(s) did you take?

________________________________________________________________

Q53 Please tell us how frequently you used some **essential oils** (include different delivery methods such as containers, diffusers, candles, massage, or other aromatherapeutic delivery methods) **to prevent catching COVID-19.** (check one answer that best describes the frequency of your use of the food category)

o Never (1)

o Daily (2)

o Weekly (3)

o Monthly (4)

Display This Question:

If Please tell us how frequently you used some essential oils (include different delivery methods su... = Daily

Or Please tell us how frequently you used some essential oils (include different delivery methods su... = Weekly

Or Please tell us how frequently you used some essential oils (include different delivery methods su... = Monthly

Q54 What type of smells/odorants?

________________________________________________________________

Display This Question:

If Please tell us how frequently you used some essential oils (include different delivery methods su... = Daily

And Please tell us how frequently you used some essential oils (include different delivery methods su... = Weekly

And Please tell us how frequently you used some essential oils (include different delivery methods su... = Monthly

Q55 What delivery method(s) do you use?

▢ containers (4)

▢ diffuser (5)

▢ candles (6)

▢ massage (7)

▢ others (8) __________________________________________________

Q56 Please tell how frequently you used some **essential oils** (include different delivery methods such as containers, diffusers, candles, massage, or other aromatherapeutic delivery methods) **to facilitate recovery from COVID-19.** (check one answer that best describes the frequency of your use of the food category)

o Never (1)

o Daily (2)

o Weekly (3)

o Monthly (4)

o Did not get sick by COVID-19 (5)

Display This Question:

If Please tell how frequently you used some essential oils (include different delivery methods such... = Daily

Or Please tell how frequently you used some essential oils (include different delivery methods such... = Weekly

Or Please tell how frequently you used some essential oils (include different delivery methods such... = Monthly

Q57 What type of smells/odorants?

________________________________________________________________

Display This Question:

If Please tell how frequently you used some essential oils (include different delivery methods such... = Daily

Or Please tell how frequently you used some essential oils (include different delivery methods such... = Weekly

Or Please tell how frequently you used some essential oils (include different delivery methods such... = Monthly

Q58 What delivery method(s) do you use?

▢ container (4)

▢ diffuser (5)

▢ candle (6)

▢ massage (7)

▢ others (8) __________________________________________________

Q59 Please tell us if there are **anything else** (for example, air freshener, perfume) that you use daily during this pandemic **to prevent catching COVID-19 or facilitate recovery from COVID-19.**

________________________________________________________________

Display This Question:

If If Please tell us if there are anything else (for example, air freshener, perfume) that you use dail... Text Response Is Not Empty

Q60 How often did you use it/them?

o Never (1)

o Daily (2)

o Weekly (3)

o Monthly (4)

Q61 Overall, are there some prescribed medicines, generic medicines, herbal medicines, home remedies, supplements, food ingredients, fluids, essential oils, or others that you think are effective in treating sickness and recovering from COVID-19 symptoms?

o Yes (1)

o No (2)

o Did not get COVID-19 (3)

o Did not try anything (4)

Display This Question:

If Overall, are there some prescribed medicines, generic medicines, herbal medicines, home remedies,... = Yes

Q62 If yes, please describe up to the top three:

o 1. What was it? (1) __________________________________________________

o 1. For what symptom? (2) __________________________________________________

o 2. What was it? (3) __________________________________________________

o 2. For what symptom? (4) __________________________________________________

o 3. What was it? (5) __________________________________________________

o 3. For what symptom? (6) __________________________________________________

Display This Question:

If Overall, are there some prescribed medicines, generic medicines, herbal medicines, home remedies,... = No

Q63 If No, is there anything that you heard that helps and you tried, but did it not work? Please describe up to three:

o 1. What was it? (1) __________________________________________________

o 1. For what symptom? (2) __________________________________________________

o 2. What was it? (3) __________________________________________________

o 2. For what symptom? (4) __________________________________________________

o 3. What was it? (5) __________________________________________________

o 3. For what symptom? (6) __________________________________________________

Q64 What year were you born?

________________________________________________________________

Q65 What is your gender?

o Male (1)

o Female (2)

o Non-binary / third gender (3)

o Prefer not to say (4)

Q66 What is your ethnicity?

________________________________________________________________

Q67 Your current location.

o City (1) __________________________________________________

o Country (2) __________________________________________________

Q68 Do you consider the answers you provided here to be strongly influenced by a specific (or several specific) country/culture(s)?

o Yes (1)

o No (2)

Display This Question:

If Do you consider the answers you provided here to be strongly influenced by a specific (or several... = Yes

Q69 If yes, which country or culture?

________________________________________________________________

Q70 Do you smoke?

o Yes (1)

o No (2)

Q71 Do you vape?

o Yes (1)

o No (2)

Display This Question:

If Do you vape? = Yes

Q72 If yes, which flavor(s) do you add?

________________________________________________________________

Q73 Did you have pre-existing health conditions pre-pandemic?

o Yes (1)

o No (2)

Display This Question:

If Did you have pre-existing health conditions pre-pandemic? = Yes

Q74 Which health conditions?

________________________________________________________________

Q75 Is there anything else that you would like to share about how best to treat Covid-19 symptoms?

o Yes (4) __________________________________________________

o No (5)

Q76 Lastly, did your eating/drinking habits change from pre-pandemic? If yes, explain. Did you stop eating certain items? Did you start eating certain items more?

o Yes, started to eat/drink more XX: Please tell me what you started to eat/drink more (4) __________________________________________________

o Yes, started not to eat/drink YY: Please tell us what you started not to eat/drink or eat/drink less (5) __________________________________________________

o No (6)

End of Block: Block 2
